# Injury-induced *Foxm1* expression in the mouse kidney drives epithelial proliferation by a cyclin F–dependent mechanism

**DOI:** 10.1172/jci.insight.175416

**Published:** 2024-06-25

**Authors:** Megan L. Noonan, Yoshiharu Muto, Yasuhiro Yoshimura, Aidan Leckie-Harre, Haojia Wu, Vladimir V. Kalinichenko, Benjamin D. Humphreys, Monica Chang-Panesso

**Affiliations:** 1 Division of Nephrology, Department of Medicine, Washington University in St. Louis School of Medicine, St. Louis, Missouri, USA.; 2 Phoenix Children’s Health Research Institute, Department of Child Health, University of Arizona College of Medicine, Phoenix, Arizona, USA.; 3 Division of Neonatology, Phoenix Children’s Hospital, Phoenix, Arizona, USA.; 4 Department of Developmental Biology, Washington University in St. Louis School of Medicine, St. Louis, Missouri, USA.

**Keywords:** Cell biology, Nephrology, Cell cycle

## Abstract

Acute kidney injury (AKI) strongly upregulates the transcription factor *Foxm1* in the proximal tubule in vivo, and *Foxm1* drives epithelial proliferation in vitro. Here, we report that deletion of *Foxm1* either with a nephron-specific Cre driver or by inducible global deletion reduced proximal tubule proliferation after ischemic injury in vivo. *Foxm1* deletion led to increased AKI to chronic kidney disease transition, with enhanced fibrosis and ongoing tubule injury 6 weeks after injury. We report ERK mediated *FOXM1* induction downstream of the EGFR in primary proximal tubule cells. We defined *FOXM1* genomic binding sites by cleavage under targets and release using nuclease (CUT&RUN) and compared the genes located near *FOXM1* binding sites with genes downregulated in primary proximal tubule cells after *FOXM1* knockdown. The aligned data sets revealed the cell cycle regulator cyclin F (*CCNF*) as a putative *FOXM1* target. We identified 2 cis regulatory elements that bound *FOXM1* and regulated *CCNF* expression, demonstrating that *Ccnf* is strongly induced after kidney injury and that *Foxm1* deletion abrogates *Ccnf* expression in vivo and in vitro. Knockdown of *CCNF* also reduced proximal tubule proliferation in vitro. These studies identify an ERK/FOXM1/CCNF signaling pathway that regulates injury-induced proximal tubule cell proliferation.

## Abstract 2

After acute kidney injury, upregulation of FoxM1 in the proximal tubule drives epithelial proliferation through the activation of the atypical cyclin-dependent kinase cyclin F.

## Graphical abstract

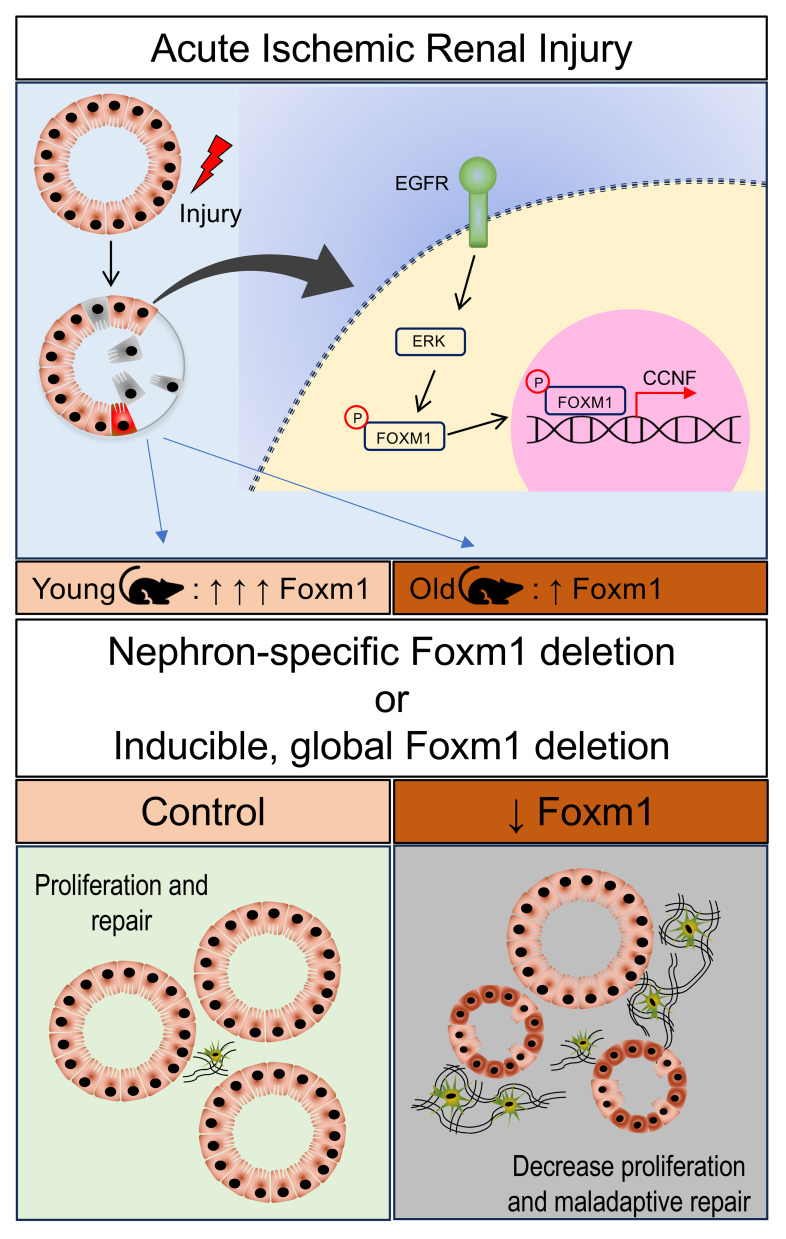

## Introduction

A central feature of successful repair after acute kidney injury (AKI) is epithelial proliferation. We and others have shown that surviving tubular epithelial cells dedifferentiate and proliferate to repair injured proximal tubule (1–4). While some of the signaling events regulating this process are known (5), many others remain unknown. In the inpatient clinical setting, AKI is encountered frequently in elderly patients, and recent studies have demonstrated that patients aged 65 and older are at higher risk of developing AKI and that subsequently some will progress to chronic kidney disease (CKD) (6–8). The cellular and molecular features of the AKI to CKD transition are beginning to be characterized, and an important aspect of this transition is that some epithelial cells undergo “failed” or “maladaptive” repair, adopting a pro-inflammatory and profibrotic phenotype that is hypothesized to drive the AKI to CKD transition (9–13). Whether defects in the early epithelial proliferative response may promote the AKI to CKD transition remains undefined.

We previously employed translational profiling of injured proximal tubules and identified the transcription factor *Foxm1* as strongly upregulated in acutely injured proximal tubule cells and showed that *Foxm1* drives proliferation in a cell culture model (3). A subsequent study by Sinha et al., using the putative FOXM1 inhibitor thiostrepton, also implicated *Foxm1* as a regulator of tubular epithelial repair following AKI in vivo (14). It should be noted, however, that thiostrepton has potent off-target effects, including inhibition of the 20s proteasome (15, 16) and arrest of mitochondrial protein synthesis (17). In fact, FOXM1 inhibition has been proposed to be a class effect of proteasomal inhibitors in general (18), and some have concluded that thiostrepton cannot be used to identify cellular consequences of *FOXM1*-DNA binding (19). These two studies linking *Foxm1* to renal tubular epithelial repair were the first to find a role for *Foxm1* in kidney injury and repair, as prior studies had looked at *FOXM1* only in the context of renal cell carcinoma (20, 21). Since then additional studies have been published describing a role for *Foxm1* in renal fibrosis (22), diabetic nephropathy (23), and polycystic kidney disease (24). *Foxm1* regulates proliferation after injury in other organs as well, including lung (25), liver (26, 27), and pancreas (28). *Foxm1* is upregulated in a variety of cancers and represents a therapeutic target (29). Intriguingly, *FOXM1* expression is repressed with aging in various organs in both mice (30, 31) and humans (32, 33). Related to this, it was recently shown that pulses of *Foxm1* overexpression in both naturally aged mice and progeroid mice led to an increase in health span and amelioration of some aging features (34). The implication of this recent work is that suppression of *Foxm1* responses with age is not simply associated with aging but is actually driving the aging process. This raises the questions of whether there is differential expression of *Foxm1* during injury with aging and whether the observation that the acutely injured aged mouse kidney is characterized by reduced proliferative capacity compared with young kidneys (35–37) is due to reduced *Foxm1* expression.

Given the apparent importance of *Foxm1* to proximal tubule repair after injury, in the present study we first sought to rigorously evaluate whether *Foxm1* drives proliferation using precise genetic models, particularly given the off-target properties of thiostrepton. We then sought to clarify the signaling intermediate between EGFR activation and FOXM1 activation. Finally, we identified *FOXM1*-DNA binding sites genome-wide through cleavage under targets and release using nuclease (CUT&RUN) and combined this with bulk RNA sequencing (RNA-Seq) from primary proximal tubule cultures subjected to *FOXM1* siRNA knockdown. We leveraged the aligned data sets to identify a shortlist of candidate *FOXM1* targets in proximal tubule. Among these we identified cyclin F (*CCNF*) as a putative mediator of *FOXM1*-dependent epithelial proliferation, which we validated by gene expression, *Foxm1* knockout, and *CCNF* knockdown.

## Results

### Foxm1 and its target genes are specifically induced in the proximal tubule after ischemia/reperfusion injury.

Having previously identified *Foxm1* as upregulated in injured proximal tubule by translational profiling (3), we verified this observation by interrogating a more recent single-nucleus RNA-Seq data set from injured mouse kidney. This revealed strong upregulation of *Foxm1* in the S3 segment of the proximal tubule with maximal expression at 48 hours after injury (Supplemental Figure 1A; supplemental material available online with this article; https://doi.org/10.1172/jci.insight.175416DS1). Lower intensity upregulation was observed in the S2 and S1 segments. This peak of expression at 48 hours correlates with the maximum proliferative phase after injury (1, 38). We validated the expression of *Foxm1* by ISH in injured kidney tissues (Supplemental Figure 1B). We also verified that known direct targets of *Foxm1* related to cell cycle (*Aurkb*, *Ccnb1*, and *Plk1*) and DNA repair (*Brca1*, *Birc5*, and *Rad51*) were also strongly upregulated in injured proximal tubule, with peak expression at 48 hours after injury (Supplemental Figure 1C).

### Nephron-specific Foxm1 deletion reduces proximal tubule proliferation after ischemic injury.

**Figure 1:**
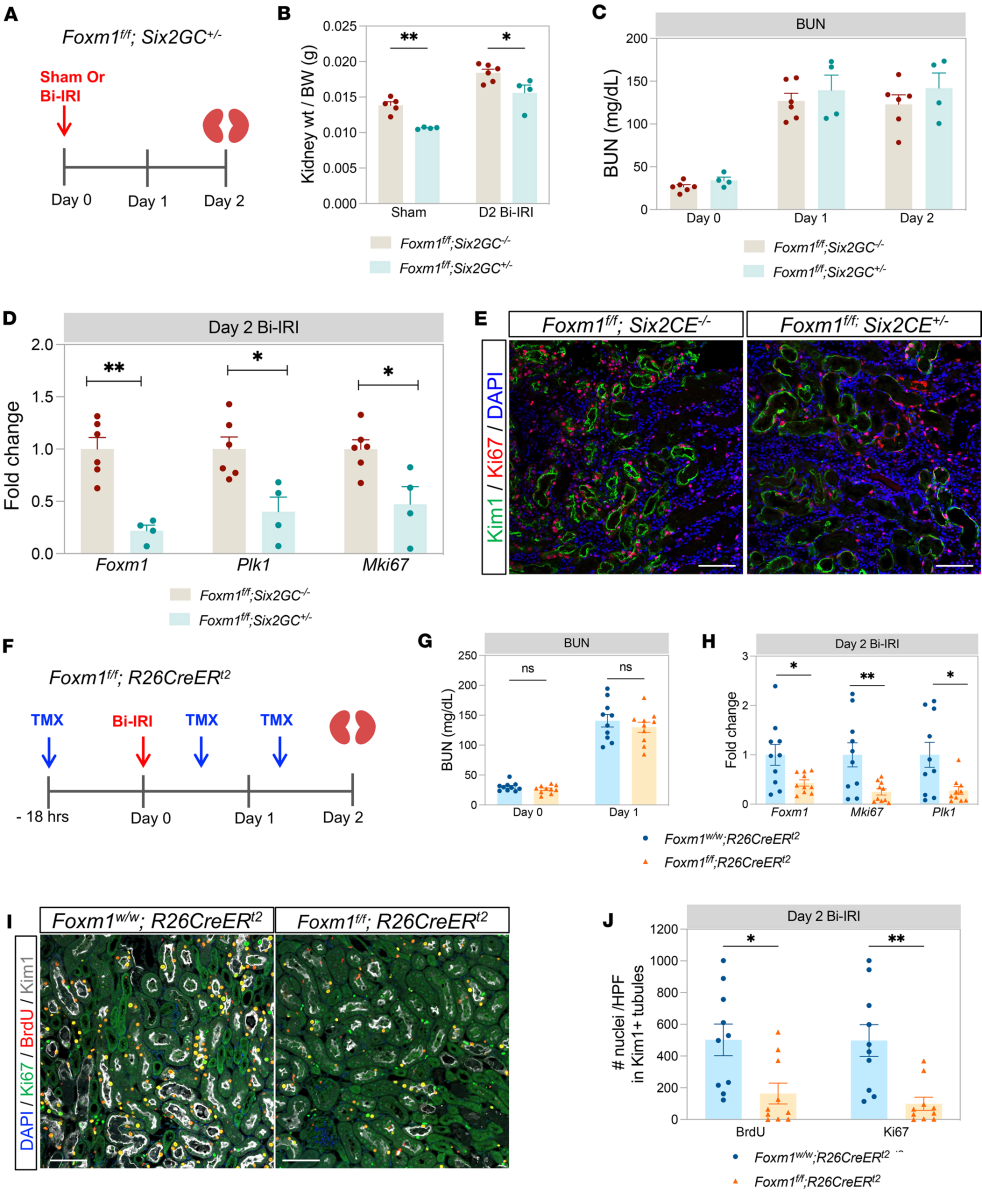
Nephron-specific and inducible, global deletion model to study *Foxm1*. (**A**) Experimental protocol. Mice underwent sham or bilateral ischemia/reperfusion injury (Bi-IRI). Kidneys were harvested at day 2 after surgery. (**B**) Graph showing kidney weights in relation to body weights. (**C**) BUN measurement at day 0, 1, and 2. (**D**) qPCR showing *Foxm1*, *Plk1*, and *Mki67* mRNA expression at 2 days after Bi-IRI. (**E**) Immunostaining for the proliferation marker Ki67 in kidney sections on day 2 after Bi-IRI. (**F**) Tamoxifen (TMX) timing protocol. (**G**) BUN at day 0 and day 1 after Bi-IRI. (**H**) qPCR showing *Foxm1*, *Mki67*, and *Plk1* mRNA expression 2 days after Bi-IRI in *Foxm1-*deleted kidneys vs. controls. (**I**) Immunofluorescence staining for Ki67, bromodeoxyuridine (BrdU), and KIM1 in kidney sections at day 2 after Bi-IRI from *Foxm1*-deleted vs. control mice. (**J**) Quantification of the number of BrdU- and Ki67-positive nuclei in tubules expressing KIM1 in the 2 different groups. HPF, high-power field. For **B**–**D**, *n* = 4–5 mice per group in sham and 4–6 for Bi-IRI. For **G**–**J**, *n* = 10 mice per group. Scale bar, 100 μm. *P < 0.05, **P < 0.01, by 2-tailed Student’s *t* test.

To test whether injury-induced *Foxm1* drives proximal tubule proliferation in vivo, we next sought to generate a tubule-specific *Foxm1*-knockout model. We first crossed the *Six2-eGFPCre* driver (39), which is active in nephron progenitors during development, against a *Foxm1* conditional allele (40). We used knockout (*Foxm1^fl/fl^* *Six2GC*^+/–^) and control (*Foxm1^fl/fl^* *Six2GC^–/–^*) littermates and subjected them to sham surgery or bilateral ischemia/reperfusion injury (Bi-IRI), collecting the kidneys at day 2 after injury (Figure 1A). Bi-IRI was performed for 18.5 minutes in 8- to 12-week-old male mice. We unexpectedly observed a statistically significant kidney weight difference in control versus mutant mice, with mutant kidneys weighing approximately 10% less than control kidneys (Figure 1B). We determined that this did not reflect a developmental phenotype resulting from *Foxm1* knockout during tubulogenesis because kidneys from the parental *Six2GC* lacking the *Foxm1*-floxed allele also had smaller kidneys (Supplemental Figure 2A). Because we could detect no proteinuria and histologically the *Foxm1^fl/fl^ Six2GC^–/–^* kidneys were normal (Supplemental Figure 2B), we proceeded with Bi-IRI surgery. In the groups that underwent Bi-IRI, the blood urea nitrogen (BUN) elevation was comparable among the 2 genotypes (Figure 1C), reflecting an equivalent degree of acute injury. There was an 80% decrease in *Foxm1* mRNA expression in the kidneys from the nephron-specific *Foxm1* deletion (Figure 1D), indicating successful *Foxm1* tubule-specific deletion. We observed downregulation of the proliferation marker Ki67 and the *Foxm1* target *Plk1* mRNA (Figure 1D) in the *Foxm1* knockout. This was accompanied by reduction in Ki67 expression 2 days after Bi-IRI in *Foxm1*-knockout kidneys as assessed by immunofluorescence (Figure 1E). Taken together, these results suggest that induction of *Foxm1* in injured proximal tubule does drive epithelial proliferation, albeit with the caveat that the *Six2GC* driver is associated with smaller kidneys.

### Inducible global deletion of Foxm1 recapitulates the decreased proliferative response after ischemic injury.

We next sought to assess whether the lack of tubular *Foxm1* and associated reduced proximal tubule proliferation might exacerbate the delayed development of renal fibrosis after AKI, the so-called AKI to CKD transition. Because smaller kidneys may reflect reduced nephron endowment, which could confound our ability to model the AKI to CKD transition, we developed a different conditional *Foxm1* deletion model. We chose not to use the *Slc34A1-CreERt2* proximal tubule–specific driver because *Foxm1* expression is strongest in the S3 segment, and that particular Cre driver acts primarily in the S1 and S2 segments (2). We therefore crossed *Foxm1^fl/fl^* mice with the *R26CreER^t2^* mice to generate bigenic *Foxm1^fl/fl^ R26CreER^t2+/–^* progeny. *R26CreER^t2^* mice carrying the *Foxm1* wild-type allele served as the control group. Notably, there was no difference in kidney size between genotypes in this model.

We once again performed Bi-IRI for 18.5 minutes in male mice at 8–12 weeks of age. Tamoxifen dosing and time points for blood collection and kidney harvesting are shown in Figure 1F. The measured BUN on day 1 after injury was comparable among the 2 groups, indicating that the extent of injury was similar (Figure 1G). We verified that there was an approximately 60% decrease in *Foxm1* mRNA expression compared with controls at 48 hours after injury (Figure 1H). There were also a statistically significant decrease in *Ki67* mRNA expression in the *Foxm1*-deleted group and downregulation of direct *Foxm1* target *Plk1* as expected (Figure 1H). We also verified a decrease in Ki67 protein expression in the proximal tubule in the *Foxm1*-knockout group (Figure 1I). Quantification of Ki67 expression in injured proximal tubules (as reflected by KIM1 positivity) revealed an approximately 70% decrease in Ki67 expression in KIM1^+^ tubules (Figure 1J). We corroborated this observation by administering the thymidine analog BrdU 3 hours before sacrifice at 48 hours after injury. Quantification of the number of BrdU-positive nuclei in KIM1^+^ injured proximal tubules revealed an approximately 60% decrease in *Foxm1*-knockout mice (Figure 1J). Together, these results demonstrate that *Foxm1* drives proximal tubule proliferation in vivo after ischemic kidney injury.

### Foxm1 knockout after ischemic injury promotes the AKI to CKD transition.

**Figure 2:**
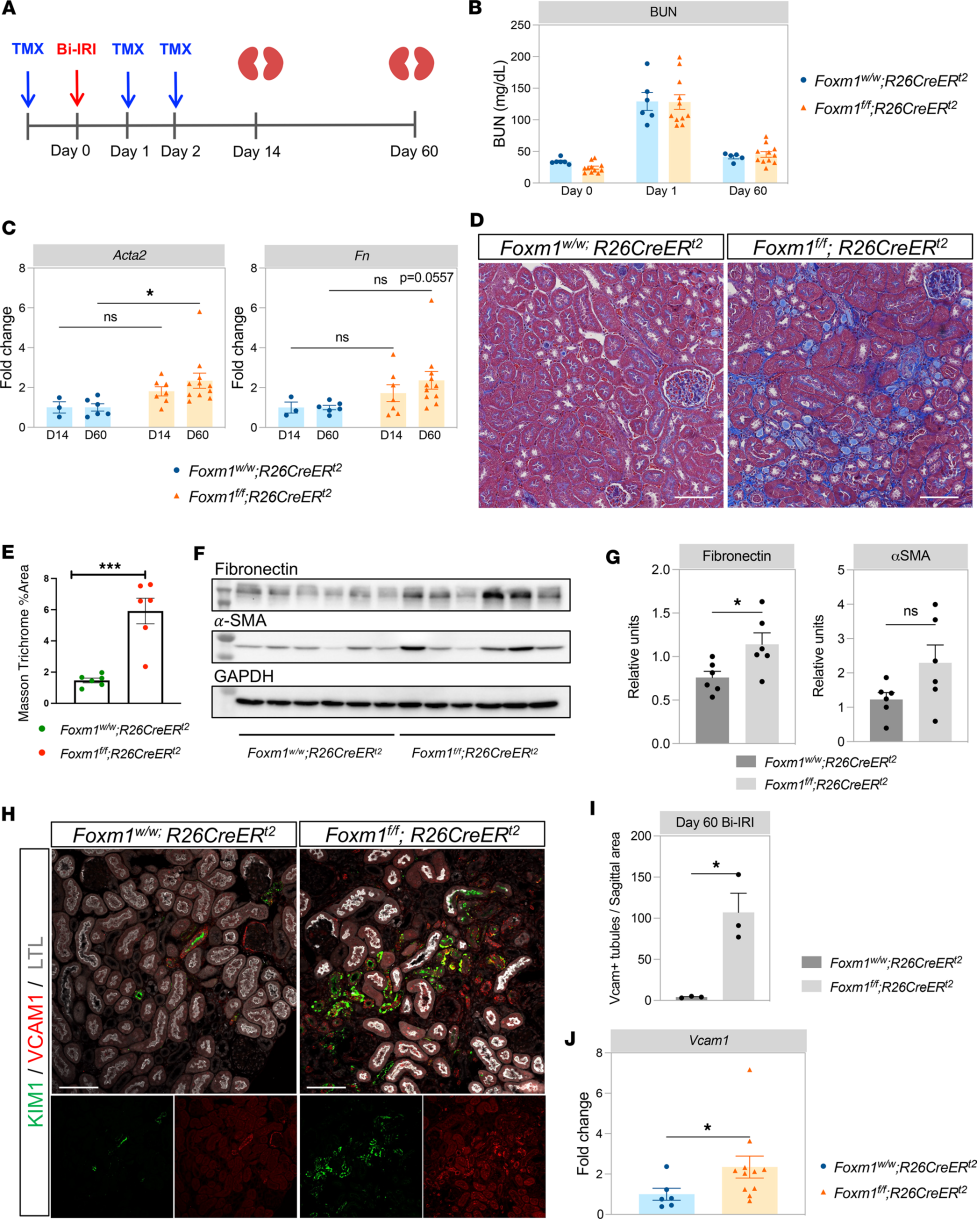
Long-term effect of *Foxm1* deletion during IRI injury. (**A**) Experimental protocol for evaluation of long-term effect of inducible *Foxm1* deletion after Bi-IRI. (**B**) BUN measurement at day 0, 1, and 60 after Bi-IRI in both groups. (**C**) qPCR for *Acta2* (α–smooth muscle actin, α-SMA) and *Fn* (fibronectin) in kidney samples from day 14 and day 60 after Bi-IRI in both groups. (**D**) Masson’s trichrome staining in kidney sections 60 days after Bi-IRI from *Foxm1* inducible, global deletion. (**E**) Quantification of collagen expression from Masson’s trichrome staining. (**F**) Western blot probing for fibronectin and α-SMA in kidney lysates at day 60 after Bi-IRI. (**G**) Densitometry of the Western blot images in **F**. (**H**) Immunostaining for VCAM1 and KIM1 in kidney sections at day 60 after Bi-IRI. (**I**) Quantification of the number of tubules expressing VCAM1 per sagittal area. (**J**) *Vcam1* expression by qPCR in kidney lysates at day 60 after Bi-IRI. For **B**–**C**, *n* = 3–7 for day 14 and *n* = 6–11 for day 60. For **E**–**H**, *n* = 6. For **I**, *n* = 3. For **J**, *n* = 6–11. Scale bar 50 μm (**D**), 100 μm (**D** and **I**). *P < 0.05, ***P < 0.001 by 2-way ANOVA with post hoc Bonferroni’s multiple comparisons tests in **C** and by 2-tailed Student’s *t* test in **E**, **G**, **I**, and **J**.

We next asked whether there were any long-term consequences of *Foxm1* deletion and reduced proliferation after injury. Specifically, we hypothesized that a blunted proliferative response might worsen the AKI to CKD transition, since proliferation of surviving epithelia is a feature of adaptive kidney repair (2). To address this, we used the same tamoxifen dosing scheme performed Bi-IRI and collected the kidneys at day 14 and 60 (Figure 2A). The degree of injury was again comparable between the groups as reflected by the similar BUN at day 1 after injury. By day 60, there was no detectable change in the BUN between the groups (Figure 2B). Since BUN is an insensitive marker of changes in kidney function, we also measured markers of fibrosis. Quantitative PCR (qPCR) analysis showed increased *Acta2* and *Fn* mRNA expression at day 60 after injury, consistent with a modestly increased AKI to CKD transition in the *Foxm1*-knockout mice (Figure 2C). Fibrosis evaluation by Masson’s trichrome staining showed increased collagen deposition in the *Foxm1*-knockout group, though this increase was patchy, and other areas of the knockout kidneys appeared histologically normal (Figure 2D). The control kidneys lacked this patchy collagen deposition. Quantification of the areas with increased collagen deposition in the knockout compared with controls revealed a 4-fold increase in trichrome staining (Figure 2, D and E). Western blot analysis showed increased fibronectin protein in the *Foxm1*-deleted group as compared with the controls (Figure 2, F and G). α-SMA protein expression trended higher in the *Foxm1*-deleted group, but this was not statistically significant, likely due to high intragroup variability (Figure 2, F and G). VCAM1 is considered a marker of failed repair in proximal tubules after injury (1, 2). We assessed the expression of VCAM1 in both control and *Foxm1*-deleted mice by immunofluorescence (Figure 2H), and there was a higher number of tubules with VCAM1 expression in the mutant mice as compared with controls (Figure 2I). Some of these VCAM1-positive tubules coexpressed KIM1, suggesting ongoing injury. The increase in *Vcam1* at the mRNA level was more modest, with a 2.3-fold increase in *Vcam1* expression in the *Foxm1*-deleted mice compared with controls (Figure 2J). Taken together, these results indicate that the loss of tubular *Foxm1* both decreases proximal tubule proliferation and modestly increases the AKI to CKD transition.

### Foxm1 induction is repressed after injury in aged mice.

**Figure 3:**
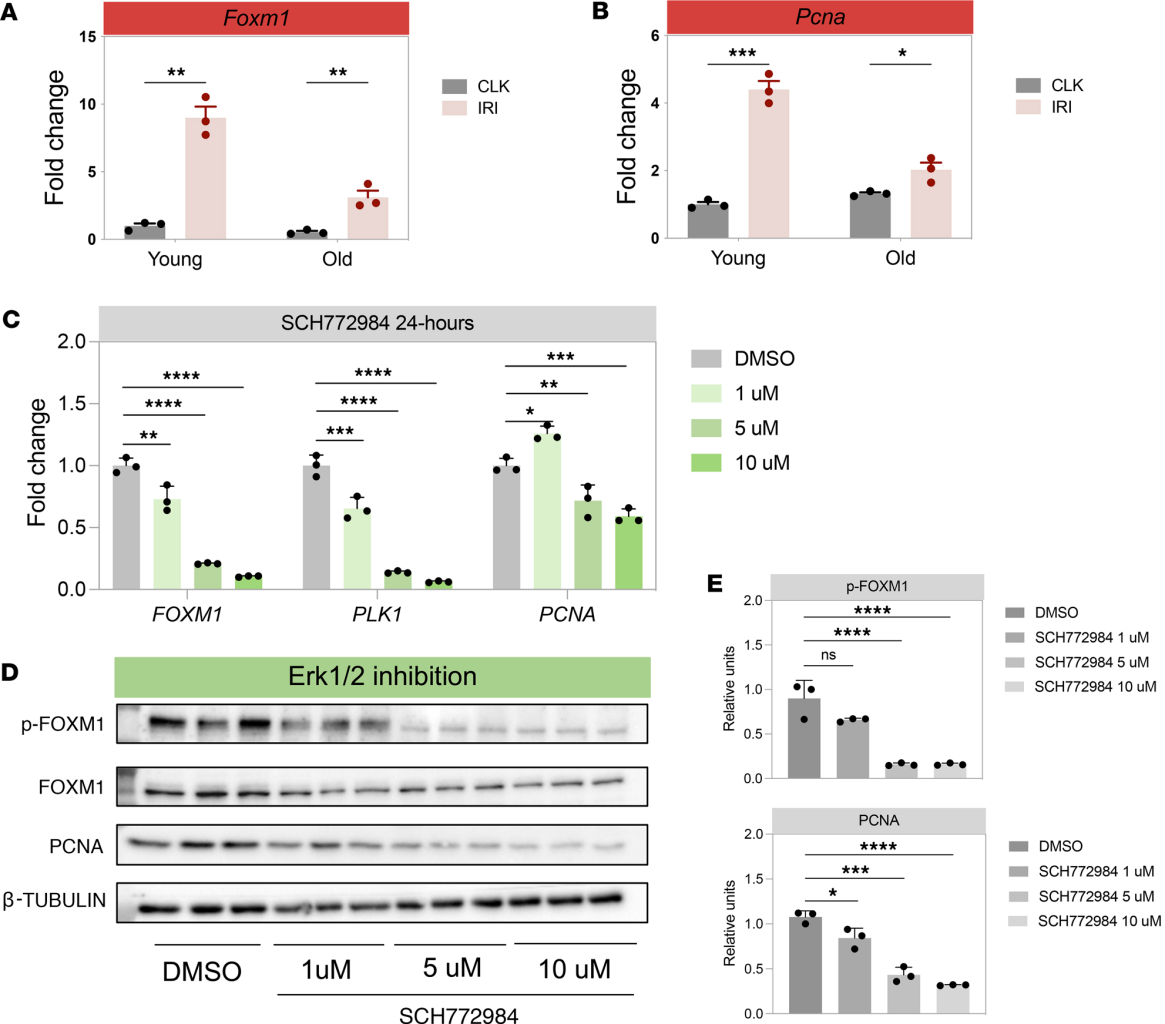
Blunted *Foxm1* upregulation in aged mouse kidney after injury and ERK signaling upstream of *FOXM1* in hRPTECs. (**A**) *Foxm1* and (**B**) *Pcna* mRNA expression in young vs. old contralateral (CLK) and injured (IRI) mouse kidneys. (**C**) *FOXM1*, *PLK1*, and *PCNA* mRNA expression by qPCR in cell lysates from hRPTECs treated for 24 hours with various doses of the ERK inhibitor SCH772984. (**D**) Western blot from cell lysates treated with SCH772984. (**E**) Densitometry of the phosphorylated FOXM1 and PCNA bands in **C**. For **A** and **B**, *n* = 3 mice per group. *P < 0.05, **P < 0.01, ***P < 0.001, by 2-tailed Student’s *t* test. For **C**–**E**, *n* = 3 replicates per group. *P < 0.05, **P < 0.01, ***P < 0.001, ****P < 0.0001 by 1-way ANOVA with post hoc Dunnett’s multiple-comparison test.

Aging is characterized by reduced proliferative potential, and *Foxm1* has been reported to be repressed with age, leading to cellular senescence in fibroblasts (33). Having established that *Foxm1* regulates proximal tubule proliferation after injury in young mice, we next investigated whether its induction might also be repressed in aged, injured kidneys, since aging is known to accelerate the AKI to CKD transition (12, 41). We performed unilateral IRI in 10-week-old and 22-month-old C57BL/6J mice for 25 minutes and harvested the kidneys 3 days after injury. While *Foxm1* mRNA was upregulated in both young and aged injured kidneys, the magnitude of upregulation was less in the injured kidneys of older mice compared with the injured kidneys of young mice (Figure 3A). Consistent with reduced upregulation of *Foxm1* in the aged kidney, there was also a more blunted expression of the proliferation maker *Pcna* in the injured kidney of aged mice as compared with the young mice, suggesting a decreased proliferative response (Figure 3B). These results suggest that aging-associated repression of *Foxm1* may partially explain worse outcomes from an episode of AKI in the elderly (42).

### The ERK pathway regulates Foxm1 activation.

We have previously identified through in vitro and in vivo studies that *Foxm1* is downstream of the EGFR (3); however, the signaling pathway between EGFR and *Foxm1* is undefined. We had also shown that human renal proximal tubular epithelial cells (hRPTECs) can be used to study EGFR/FOXM1 signaling because EGF ligand is present in the medium, and this drives *Foxm1*-dependent proliferation of this primary cell line (3). To further dissect if other ligands and receptors could induce activation of *FOXM1* in hRPTECs, we designed a series of in vitro experiments using a combination of inhibitors, ligands, and siRNA *FOXM1* knockdown. We first asked whether serum starvation has an effect on *FOXM1*. Cells were collected after 16 hours of overnight culture in renal epithelial basal media with no supplements or serum. This resulted in significant downregulation of *FOXM1*, *MKI67*, and *PLK1* mRNA expression compared with control cells (Supplemental Figure 3A). We tested several pathways and receptors that have been implicated in AKI that could potentially be involved in *FOXM1* regulation, including mTOR, TGF-β receptor, and mesenchymal-epithelial transition (MET) receptor. hRPTECs were treated with increasing doses of the mTOR inhibitor rapamycin and showed no changes in *FOXM1* mRNA expression (Supplemental Figure 3B). MET receptor signaling was inhibited by treating hRPTECs with the c-Met kinase inhibitor PF-04217903 (PF) alone or in the presence of hepatocyte growth factor (HGF). Neither PF, HGF, nor combination treatments significantly altered *FOXM1* mRNA expression. However, cells treated with HGF, either alone or in combination with PF, did upregulate *MKI67* and *PLK1* mRNA expression (Supplemental Figure 3C). To investigate the effect of TGF-β receptor inhibition, hRPTECs were treated with the selective TGF-β receptor type I/II inhibitor LY2109761 (LY) alone or in the presence of TGF-β. We did observe a modest yet significant upregulation of *FOXM1* expression in the lowest LY dose with TGF-β. However, this did not occur at higher inhibitor doses or with the inhibitor alone (Supplemental Figure 3D). This coincided with upregulated *MKI67* and *PLK1* expression under this condition. These data support that mTOR, MET receptor, or TGF-β receptor signaling does not alter *FOXM1* expression.

To further examine the role of EGF/EGFR and FOXM1 on cell proliferation, *FOXM1* expression was knocked down using siRNA (siFOXM1), and then cells were cultured in renal epithelial growth media (REGM) with and without the standard EGF component. In scrambled siRNA control groups (siScr), REGM with EGF significantly upregulated *FOXM1* expression compared with REGM without EGF (Supplemental Figure 4A). *FOXM1* expression was markedly downregulated in siFOXM1 groups and was not different between cells with and without EGF. We previously showed serum starvation alone downregulates *FOXM1* and was replicated in this experiment compared with both REGM with and without EGF. *FOXM1* expression was further downregulated in starved siFOXM1 cells. *MKI67* expression was upregulated in cells with EGF in the media (Supplemental Figure 4B). Importantly, *FOXM1* knockdown downregulated *MKI67* expression in cells with EGF and was not significantly different from siFOXM1 cells without EGF. *FOXM1* knockdown did not alter *MKI67* expression in serum-starved conditions. We also treated siFOXM1 cells with HGF and TGF-β to determine the role of *FOXM1* in the proliferative response to these factors. HGF treatment in siScr control cells modestly upregulated *FOXM1*, *PLK1*, and *MKI67* expression compared with vehicle-treated cells (Supplemental Figure 4C). *FOXM1*-knockdown cells treated with HGF had blunted *PLK1* and *MKI67* expression compared with HGF-treated siScr cells but were still significantly increased compared with vehicle-treated siFOXM1 cells. Similarly, TGF-β treatment in siScr control cells upregulated *FOXM1*, *PLK1*, and *MKI67* expression compared with vehicle treatment (Supplemental Figure 4D). *FOXM1*-knockdown cells treated with TGF-β had blunted *PLK1* and *KI67* expression compared with TGF-β–treated siScr cells but was still significantly increased compared with vehicle-treated siFOXM1 cells. These results support that *FOXM1* is mainly used by EGF/EGFR signaling to affect proliferation, whereas HGF/MET and TGF-β/TGFβR signaling utilize other factors in addition to *FOXM1*.

As a complementary approach to determine the role of EGFR and other signaling pathways in *Foxm1* activation, we performed *Foxm1* knockout in primary mouse tubular epithelial cells (mPTECs) from *Foxm1^fl/fl^* mice using AdenoCre. We achieved efficient knockout with AdenoCre treatment (Supplemental Figure 5A). We cultured mPTECs transduced with either AdenoCre or AdenoGFP (negative control) in media with or without EGF to determine the *Foxm1* response to EGF stimulation. We observed that EGF stimulation induced *Foxm1* and *Ki67* expression; however, *Foxm1* remained suppressed in *Foxm1*-knockout cells despite the presence of EGF. Another group of cells were treated with either HGF or TGF-β to test if these ligands exert an effect on *Foxm1* expression. Treatment with HGF did not induce *Foxm1* in the control group or the AdenoCre group (Supplemental Figure 5B). On the other hand, treatment with TGF-β downregulated *Foxm1* expression in the control group but had no effect in the AdenoCre group (Supplemental Figure 5C). These results indicate that in mPTECs, EGFR mediates its proliferative signal through *Foxm1*; however, c-Met receptor does not appear to be signaling through *Foxm1*. TGF-β downregulates *Foxm1*, which is perhaps expected since TGF-β is cytostatic; however, *Foxm1* was not completely suppressed, suggesting that TGF-β does not exert a strong regulatory role on *Foxm1*. Of note, in hRPTECs TGF-β appeared to induce proliferation, which is opposite to the effect noted with mRPTECs, which was decreased proliferation. The observed effect could be related to different response to the concentration of TGF-β used, as it has been described that TGF-β at lower concentration can stimulate proliferation in certain cell types (43).

We then focused on the MEK/ERK pathway as an intermediate pathway between EGFR and FOXM1 because these are known to be activated by the EGFR, and roles for ERK pathway activation in promoting kidney repair are well established (44, 45). Furthermore, it has been previously described that the Raf/MEK/MAPK pathway is necessary for the phosphorylation and nuclear translocation of FOXM1 (46). Phosphorylation of FOXM1 is temporally regulated throughout the cell cycle and is important in controlling its transcriptional activity (47, 48). We utilized the ERK inhibitor SCH772984 (49) or the MEK inhibitor U0126 (50). ERK inhibition with SCH772984 in hRPTECs caused dose-dependent decreases in *FOXM1* mRNA expression and associated decreases in *FOXM1* target *PLK1* and the proliferation marker *PCNA* at higher doses (Figure 3C). We observed a modest reduction in total FOXM1 protein levels with SCH772984 treatment (Figure 3D); however, there was a more substantial dose-dependent reduction of phosphorylated FOXM1 protein with ERK inhibition (Figure 3E). The MEK inhibitor U0126 showed more modest downregulation of *FOXM1* mRNA expression only at higher doses, with no change in *PCNA* mRNA expression (Supplemental Figure 6A). However, like ERK inhibition with SCH772984, there was also a dose-dependent decrease in phosphorylated FOXM1 with MEK inhibition (Supplemental Figure 6, B and C). The lack of effect on *PCNA* mRNA expression with the MEK inhibitor, U0126, despite phosphorylated FOXM1 downregulation, is possibly due to inhibitor off-target effects, as protective effects have been attributed to U0126 independent of its function as an MEK inhibitor (51–53). These results suggest that the EGFR regulates FOXM1 through the MEK/ERK pathway.

### Cyclin F is a direct FOXM1 downstream target.

**Figure 4:**
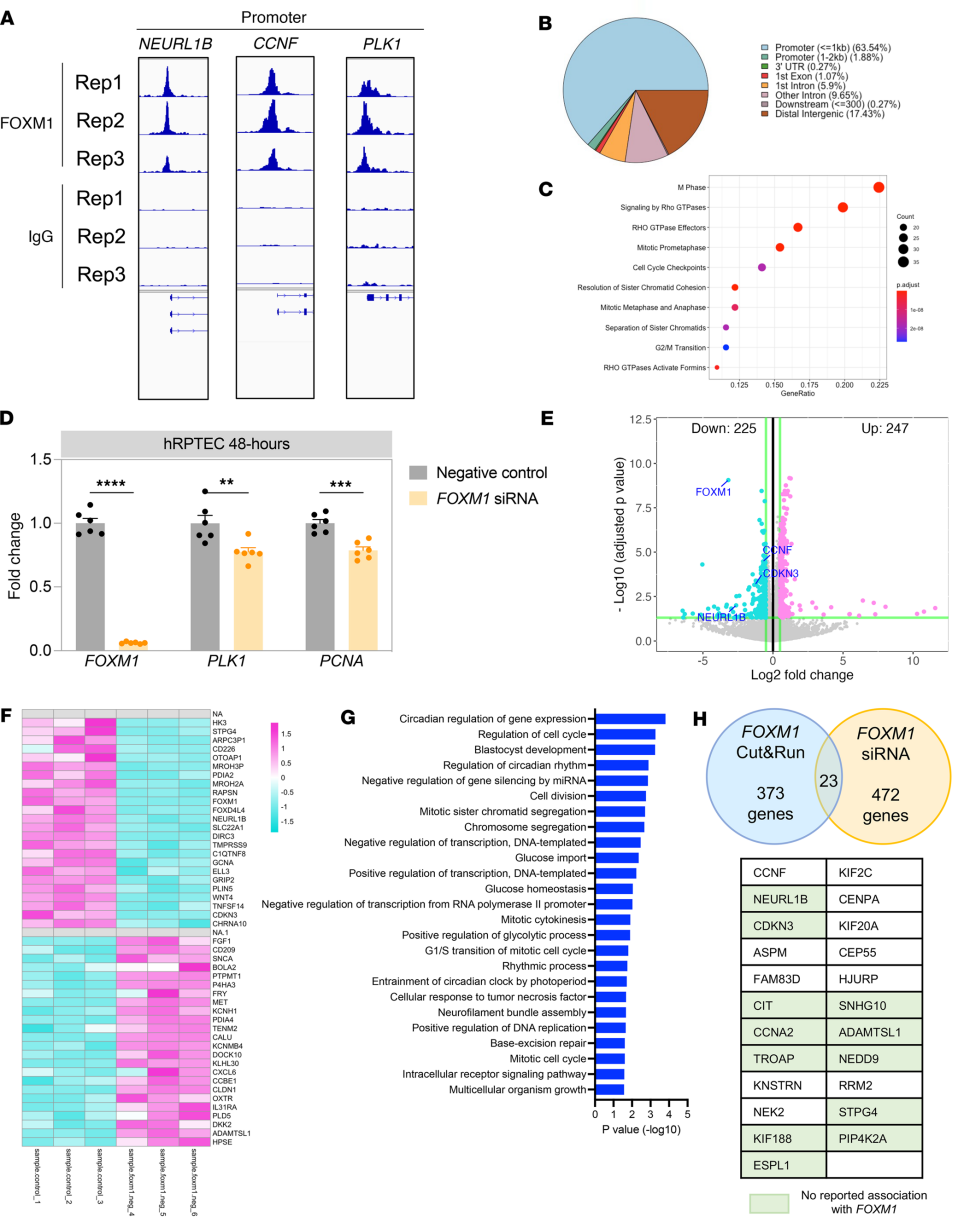
Identifying downstream targets of *FOXM1*. (**A**) CUT&RUN for *FOXM1* was performed on hRPTECs. (**B**) Genomic distribution of *FOXM1* CUT&RUN peaks. (**C**) Gene ontology analysis of the binding peaks identified. (**D**) qPCR showing *FOXM1*, *PLK1*, and *PCNA* mRNA expression after *FOXM1* siRNA treatment of hRPTECs. (**E**) Bulk RNA-Seq was performed on lysates from *FOXM1* siRNA and control siRNA-transfected hRPTECs. (**F**) Heatmap showing the top 25 upregulated genes and downregulated genes from the DEG list by comparing *FOXM1* siRNA–treated hRPTECs vs. control. (**G**) Gene ontology analysis of the DEG list after *FOXM1* siRNA treatment in hRPTECs. (**H**) Genes that overlapped in the *FOXM1* CUT&RUN and *FOXM1* bulk RNA-Seq data sets. Genes highlighted have not been reported to be associated with *FOXM1*. **P < 0.01, ***P < 0.001, ****P < 0.0001 by 2-tailed Student’s *t* test.

We next attempted to identify downstream targets of *FOXM1* regulating proximal tubule proliferation. To do this, we performed CUT&RUN sequencing (Figure 4, A and B) with an anti-FOXM1 antibody. This analysis identified 373 genes with putative *FOXM1* binding sites, including both known *FOXM1* targets and what we believe to be novel ones. *PLK1* is a well-known *FOXM1* downstream target, and it was also identified in our data set, thus validating our approach. Other genes with a strong *FOXM1* binding peak included *NEURL1B* and *CCNF*. *NEURL1B*, also known as *Neur2*, is an E3 ubiquitin ligase involved in internalization and degradation of Notch ligands, which is important in the regulation of the Notch pathway (54). *CCNF* encodes cyclin F, which is a member of the cyclin family and of the F-box protein family. Cyclin F forms a functional Skp, Cullin, F-box containing complex (or SCF complex) that mediates the ubiquitylation and degradation of proteins important for cell cycle progression and genome stability (55, 56). Gene Ontology analysis on the identified potential binding sites revealed terms consistent with the cell cycle as expected (Figure 4C).

As an orthogonal approach to validate these putative *FOXM1* targets, we performed bulk RNA-Seq in hRPTECs with *FOXM1* knockdown by siRNA treatment (Figure 4D). We reasoned direct *FOXM1* targets should be genes with *FOXM1* binding sites identified by CUT&RUN that are also downregulated after *FOXM1* knockdown. There were 452 differentially expressed genes (DEGs) after *FOXM1* knockdown (Figure 4E). Figure 4F shows the top 25 upregulated and downregulated genes. Gene ontology analysis on the DEG list was enriched by terms related to the cell cycle and interestingly also by terms related to regulation of transcription, circadian rhythm, and glucose (Figure 4G). We identified 23 genes that were common between the *FOXM1* siRNA and the CUT&RUN gene lists, including 12 genes not previously reported as *FOXM1* targets (Figure 4H). *CCNF* and *NEURL1B* were both included in this list. We decided to focus on *CCNF*, as it has been described to have roles in cell cycle regulation in other tissues, even though it has not been described in kidney.

### Cis regulatory elements containing FOXM1 binding sites regulate Ccnf expression.

**Figure 5:**
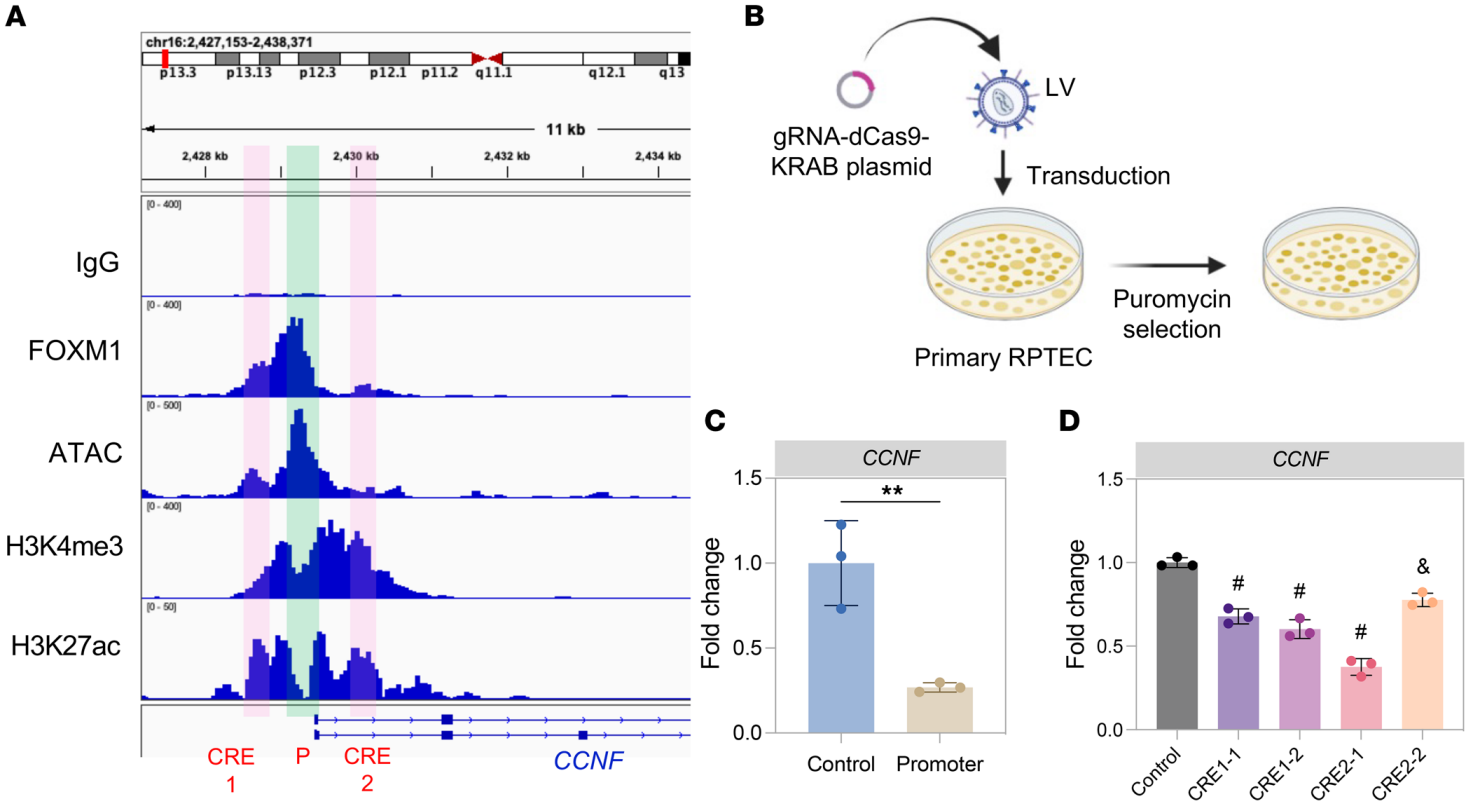
Targeting potential *FOXM1* binding sites for *CCNF* regulation using CRISPRi. (**A**) Representative tracks showing *FOXM1* CUT&RUN enrichment at the *CCNF* gene, including a negative control (no primary antibody). Tracks were aligned with CUT&RUN for histone H3 lysine 4 trimethylation (H3K4me3) and histone H3 lysine 27 acetylation (H3K27ac) and ATAC in hRPTECs to identify potential regulatory sites to target by CRISPRi. (**B**) Schematic of CRISPRi approach. Created using BioRender.com. (**C**) *CCNF* mRNA expression after targeting a potential promoter binding site by CRISPRi. (**D**) *CCNF* mRNA expression using 2 different single guide RNAs (sgRNAs) targeting potential enhancer site 1 (CRE1 in **A**) or enhancer site 2 (CRE2 in **A**). *n* = 3 replicates per experiment. **P < 0.01, ^&^*P* < 0.001, ^#^*P* < 0.0001 by 2-tailed Student’s *t* test in **C** or 1-way ANOVA with post hoc Dunnett’s multiple-comparison test in **D**.

Having identified cyclin F as a direct target of *FOXM1* both by CUT&RUN and by *FOXM1* silencing, we next sought direct evidence for *FOXM1* regulation of *CCNF* gene expression. Our strategy was to identify putative cis regulatory elements (CREs) regulating *CCNF* expression containing *FOXM1* binding motifs, to silence these using CRISPR interference (CRISPRi), and to analyze the effect on *CCNF* expression. As a positive control, we identified the *CCNF* promoter from our CUT&RUN data set, which also contained a *FOXM1* binding site (Figure 5A, green bar). Closing the *CCNF* promoter by CRISPRi caused a 73% decrease in *CCNF* mRNA expression (Figure 5, B and C). We next aligned our *FOXM1* CUT&RUN data set with data sets from ATAC-Seq and CUT&RUN for H3K4me3 and H3K27ac in hRPTECs to identify active chromatin (57, 58). We identified 2 regions — one proximal and another region distal to the *CCNF* promoter — with *FOXM1* binding peaks that aligned with active chromatin peaks, suggestive of CREs (Figure 5A, pink bars). We designed single guide RNAs (sgRNAs) to target these 2 regions by CRISPRi and observed a decrease in *CCNF* mRNA expression of 30%–40% when targeting E1 and 25%–60% when targeting E2. These results verify that these *FOXM1* binding site–containing CREs positively regulate *CCNF* expression (Figure 5D). The first CRE (E1) could be an enhancer given the predominant H3K27ac peak and relatively low H3K4me3 peak. The second CRE (E2) displayed both an H3K4me3 peak and an H3K27ac peak that aligned with a distal small *FOXM1* peak outside the TSS. Extrapolating from the candidate CRE classification used in Encode (59), this CRE perhaps may be a poised canonical promoter, or a noncanonical promoter-like element or an element with other functions around a canonical promoter with a high H3K4me3 signal.

### Evidence that Ccnf directly regulates proximal tubular proliferation.

**Figure 6:**
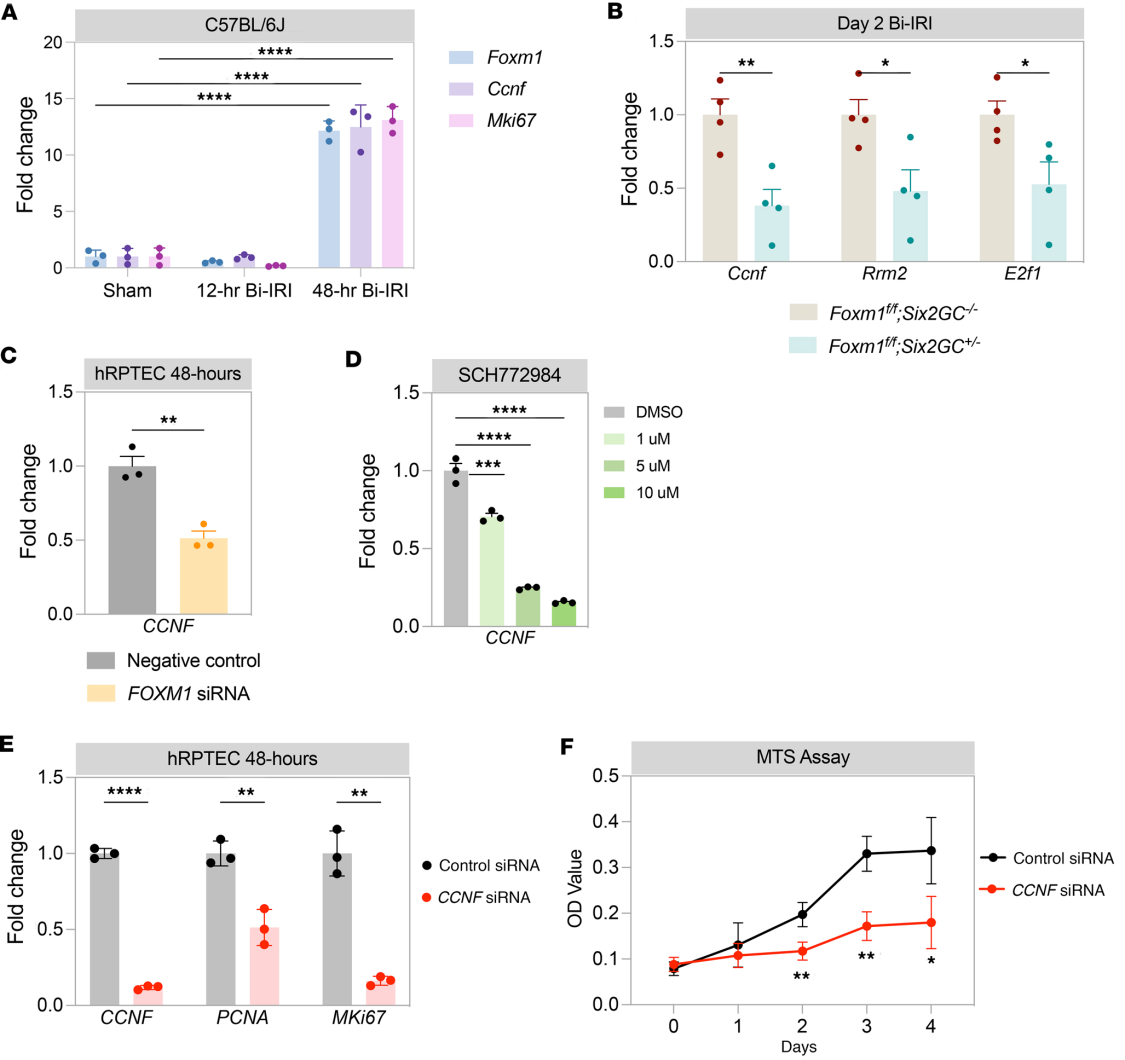
*CCNF* is downstream of *FOXM1* and regulates cell proliferation. (**A**) *Foxm1*, *Ccnf*, and *Mki67* mRNA expression in sham and injured kidneys from C57BL/6J mice at 12 hours and 48 hours after ischemia. (**B**) *CCNF*, *Rrm2*, and *E2f1* mRNA expression by qPCR in kidney lysates from nephron-specific *Foxm1* deletion 2 days after Bi-IRI. qPCR for *CCNF* in cell lysates from hRPTECs after *FOXM1* knockdown via siRNA (**C**) after treatment with ERK inhibitor SCH772984 (**D**). (**E**) qPCR in cell lysates from hRPTECs harvested 2 days after transfection with *CCNF* siRNA or control siRNA. (**F**) 3-(4,5-dimethylthiazol-2-yl)-5-(3-carboxymethoxyphenyl)-2-(4-sulfophenyl)-2H-tetrazolium (MTS) assay of hRPTECs transfected with either *CCNF* siRNA or control siRNA. *P < 0.05, **P < 0.01, ***P < 0.001, ****P < 0.0001 by 2-tailed Student’s *t* test in **A**–**C** and **E**; 1-way ANOVA with post hoc Dunnett’s multiple-comparison test in **D**; and 2-way ANOVA with post hoc Bonferroni’s multiple comparisons tests in **F**.

We evaluated *Ccnf* expression after IRI in C57BL/6J mice and found it to be upregulated at 48 hours after injury along with proliferation genes *Foxm1* and *Mki67* (Figure 6A). If *Foxm1* regulates proximal tubule proliferation by a cyclin F–dependent mechanism, then we would expect reduced *Ccnf* expression after injury in our *Foxm1*-knockout model. In injured kidneys from *Foxm1^fl/fl^ Six2GC^+/–^* mice, we observed an approximately 60% reduction in *Ccnf* expression compared with controls at 2 days after Bi-IRI (Figure 6B). *Rrm2* and *E2f1*, which are known downstream targets of *Ccnf*, were also downregulated (Figure 6B) in the *Foxm1*-deleted mice. We could also demonstrate that *FOXM1* knockdown in hRPTECs reduced *CCNF* mRNA by approximately 50% (Figure 6C). There was also reduced expression of *CCNF* in the hRPTECs treated with the ERK inhibitor (Figure 6D), as we would expect since ERK regulates FOXM1. These results suggest an injury-induced EGFR/ERK/FOXM1/CCNF signaling axis in the proximal tubule. Finally, we sought direct evidence that *CCNF* regulates proliferation in hRPTECs. Indeed, siRNA knockdown of *CCNF* (~90% knockdown) in hRPTECs (Figure 6E) led to a significant reduction in proliferation marker expression (Figure 6E). Consistent with this, direct measurement of hRPTEC proliferation after *CCNF* knockdown verified a strong reduction in cell proliferation (Figure 6F).

## Discussion

We draw 4 conclusions from the current study. First, using 2 inducible deletion models, we demonstrate that *Foxm1* regulates injury-induced proximal tubule proliferation in vivo, consistent with our prior in vitro cell culture work (3). Second, the absence of tubular *Foxm1* modestly exacerbates the AKI to CKD transition at late time points after injury in our mouse model of moderate AKI. Third, we show that EGFR drives FOXM1 activation primarily via ERK activity. Last and most important, using CUT&RUN and *FOXM1*-knockdown strategies, we identify cyclin F as a direct target of *Foxm1* in kidney and a key mediator of injury-induced proximal tubule cell proliferation.

ERK is known to be activated by IRI in mice, where it regulates tubular epithelial proliferation (44). However, the mechanism by which ERK activates cell cycle progression is undefined. Our results here indicate that ERK regulates FOXM1 expression in the injured proximal tubule and that *FOXM1* itself induces expression of cyclin F, which drives cell proliferation. We propose an EGFR/ERK/FOXM1/CCNF axis driving proximal tubule proliferation after acute injury.

Cyclin F is unique in that it does not bind cyclin-dependent kinases as do the other cyclins. Cyclin F belongs to the F-box protein family of substrate recognition receptors that recruit proteins to the SCF E3 ligase. SCF ligases mediate the ubiquitylation and degradation cell cycle inhibitors (60, 61). A recent report found that the retinoblastoma (RB) family of proteins, which repress cell cycle gene expression and inhibit proliferation, are substrates of the SCF-cyclin F E3 ligase (55). This suggests the hypothesis that in the proximal tubule, cyclin F regulates cell proliferation by ubiquitination of RB proteins, whose presence normally inhibits cell proliferation.

Our analysis revealed putative CREs containing *FOXM1* binding sites, which, when closed by CRISPRi, reduced *CCNF* expression. Furthermore, *FOXM1* siRNA knockdown downregulated *CCNF* expression, suggesting direct regulation of *CCNF* by *FOXM1*. To our knowledge, our study is the first to describe a role for *CCNF* in proximal tubule proliferation. Only one other study thus far has established *CCNF* as a direct target of *FOXM1* (62). In that study, the authors demonstrated that *CCNF* is transcriptionally activated by *FOXM1* in an ovarian cancer cell line.

Our findings may have relevance for the increased susceptibility to AKI and the AKI to CKD transition with age. The exact biological and molecular mechanisms underlying this increased susceptibility are poorly defined. In the present study, we identified that the aged mouse kidney has a blunted upregulation of *Foxm1* upon injury, which may explain the decreased tubular epithelial proliferation in aged mice observed in previous studies (35–37). Both of our mouse models suggest that decreased proliferation due to *Foxm1* deletion in the acute period led to upregulation of fibrosis markers over time. Furthermore, mutant mice had increased expression of VCAM1 in proximal tubules, and VCAM1 has been shown to label a population of tubules with maladaptive repair (9, 57). Loss of *Foxm1* in other organs or cell types has also been found to lead to development of fibrosis. For instance, in cardiomyocytes *Foxm1* loss leads to cardiac fibrosis (63), and in Clara cells, its loss during development leads to peribronchial fibrosis (64). Our findings contrast with those from other studies describing that *Foxm1* inhibition ameliorates renal fibrosis (22) and lung fibrosis (65). The study by Wang et al. indicates that *Foxm1* downregulation suppressed the epithelial-mesenchymal transition (EMT) and lessened the expression of fibrotic markers in the kidney. However, EMT as a driver of renal interstitial fibrosis is an area of intense debate (66). In fact, we have previously demonstrated that kidney epithelial cells do not differentiate into myofibroblasts in vivo, and therefore there is no evidence of EMT taking place, at least in vivo, or contributing to renal fibrosis (67). In light of our prior findings regarding EMT, it is difficult to reconcile a beneficial effect for *Foxm1* based on downregulation of EMT. Furthermore, thiostrepton was used to induce *Foxm1* downregulation, and it is possible that off-target effects are responsible for their observations, since thiostrepton off-target effects have been previously described (19). The diversity of outcomes with *Foxm1* deletion indicate that *Foxm1* may have a cell-specific and context-specific role, as there have been diverging roles for *Foxm1* within the same organ. For instance, one study showed that *Foxm1* deletion in fibroblasts ameliorates lung fibrosis (65); however, another study showed that *Foxm1* deletion in macrophages appears to promote lung fibrosis (68). Despite opposing events (repair vs. fibrosis), one common process in both appears to be proliferation, and *Foxm1* as a cell cycle regulator may play a role in both. It is likely that during injury when mitogenic signals are trying to repair, *Foxm1* is beneficial, but it may be detrimental if the cells that are upregulating its expression are responding to some pathogenic cues. Therefore, *Foxm1* probably has a context-dependent role.

In summary, our observations suggest that decreased tubular epithelial proliferation can lead to unsuccessful repair and consequently an AKI to CKD transition. In the context of aging, decreased *Foxm1* expression after injury may therefore also lead to increased AKI to CKD transition.

## Methods

### Sex as a biological variable.

Our study exclusively examined male mice, as female mice are much more resistant to IRI (69).

### Animals.

Our study examined male mice because male animals are more susceptible to IRI than females. All mouse experiments were performed according to the animal experimental guidelines issued by the Animal Care and Use Committee at Washington University in St. Louis. *Foxm1^fl/fl^* mice have been previously described (40). *Rosa26CreER^t2^* (stock 008463), *Six2GC* (stock 009606), and C57BL/6J (stock 000664) mouse lines were purchased from Jackson Laboratory. These mouse lines have a mixed genetic background. We crossed *Foxm1^fl/fl^* mice with *Foxm1^fl/fl^ Six2GC^+/–^* mice. The litter from this cross was used for the studies, with the *Foxm1^fl/fl^ Six2GC^–/–^* littermates serving as controls. For the inducible, global *Foxm1* deletion mouse model, we crossed *Foxm1^fl/wt^ R26CreERT2^+/–^* with *Foxm1^fl/wt^ R26CreERT2^+/–^* to generate study mice (*Foxm1^fl/fl^ R26CreERT2^+/–^*) and control mice (*Foxm1^wt/wt^ R26CreERT2^+/–^*).

### Surgery.

For Bi-IRI, 8- to 12-week-old male mice were anesthetized with isoflurane, and buprenorphine SR was administered for pain control. Body temperature was monitored and maintained at 36.5°C–37.5°C throughout the procedure. Bilateral flank incisions were made, and the kidneys were exposed. Ischemia was induced by clamping the renal pedicle with a nontraumatic microaneurysm clamp (Roboz) for 18.5 minutes. The clamps were removed at the time of completion. The peritoneal layer was closed with absorbable suture, and the flank incisions were closed with wound clips. For the sham procedure, the same procedure was undertaken except for omitting the clamping step.

### BUN measurement.

BUN measurement was done using the QuantiChrom Urea Assay kit (BioAssay Systems DIUR-100) as per the manufacturer’s protocol.

### Real-time PCR.

Kidney tissue was snap-frozen in liquid nitrogen at the time of harvesting. RNA was extracted using the Direct-zol Miniprep Plus kit (Zymo) following the manufacturer’s instructions. The extracted RNA (600 ng) was reverse-transcribed using the High-Capacity cDNA Reverse Transcription kit (Life Technologies, Thermo Fisher Scientific). Quantitative real-time PCR was done using the iTaq Universal SYBR Green Supermix (Bio-Rad). Expression levels were normalized to GAPDH, and data were analyzed using the 2-ΔΔCt method. Primers used are listed in Supplemental Table 1.

### Tissue preparation and histology.

After euthanasia, mice were perfused via the left ventricle with ice-cold PBS. Kidneys were harvested and the capsule was removed. They were fixed in 4% paraformaldehyde on ice for 1 hour, then incubated in 30% sucrose at 4°C overnight. The next day, tissues were embedded in OCT medium (Sakura Finetek). Kidney sections were cut at 6 μm and mounted on Superfrost slides (Thermo Fisher Scientific). Immunofluorescence staining was performed as follows. Kidney sections were washed with 1× PBS for 10 minutes and permeabilized with 0.25% Triton X-100 for 10 minutes. The tissue sections were blocked with 5% BSA in PBS for 1 hour. Primary antibodies were incubated for 1 hour at room temperature, and sections were rinsed with 1× PBS for 5 minutes 3 times. Secondary antibodies (1:200) were incubated for 1 hour at room temperature and rinsed with 1× PBS for 5 minutes 3 times. DAPI was used for counterstaining. The following antibodies were used: Kim-1 (AF1817, R&D Systems, Bio-Techne), Ki67 (14-5698, eBioscience, Thermo Fisher Scientific), α-SMA (MilliporeSigma F3777), Col1α1 (Southern Biotech 1310-01), PDGFR-β (eBioscience, Thermo Fisher Scientific, 16-1402), VCAM1 (Abcam ab134047), BrdU (Abcam ab6326), and Biotinylated-LTL (Vector Laboratories B-1325). Secondary antibodies were Alexa Fluor 488, Cy3, Cy5, or Streptavidin-Cy5 conjugated (Invitrogen, Thermo Fisher Scientific). For BrdU staining, paraffin sections were used. Antigen retrieval was performed using the citrate-based antigen unmasking solution (Vector Laboratories H-3300) and pressure cooker treatment.

### Histology quantification.

Collagen I fiber deposition was quantified using a published protocol using ImageJ (NIH) (70). Quantification was performed by taking bright-field images in random areas (original magnification, ×200; *n* = 5–6 per kidney) for each mouse. For VCAM1 quantification, tubules with positive VCAM1 staining were quantified per sagittal area for each mouse by taking images at ×40 original magnification (*n* = 3–4) to encompass most of the kidney area.

### Western blotting.

Kidney tissue was snap-frozen in liquid nitrogen upon harvesting. Tissue was homogenized in RIPA lysis buffer containing protease and phosphatase inhibitors (Roche). Protein concentration was measured using the BCA assay (Pierce, Thermo Fisher Scientific). For hRPTECs, cells were washed with 1× PBS and lysates prepared in RIPA buffer with protease and phosphatase inhibition. Using 10% polyacrylamide gel, 10–20 μg of protein was separated by SDS electrophoresis and transferred to an Immobilon PVDF membrane (MilliporeSigma). Each membrane was blocked with 5% milk in TBS with Tween (TBST) and probed overnight at 4°C with the primary antibody. After the membrane was washed with TBST, it was incubated for 1 hour at room temperature with HRP-conjugated secondary antibody (Dako P0448). The membrane was developed using the ECL detection system (GE Healthcare, now Cytiva). Primary antibodies were Foxm1 (Cell Signaling Technology 5436S), phospho-Foxm1 (Cell Signaling Technology 14655), PCNA (Cell Signaling Technology 13110S), MEK1/2 (Cell Signaling Technology 9122), phospho-MEK1/2 (Cell Signaling Technology 9121), ERK 1/2 (Cell Signaling Technology 9102), phospho-ERK 1/2 (Cell Signaling Technology 9101), α-SMA (Abcam ab5694), fibronectin (Abcam ab23750), β-tubulin (Proteintech 66240-1), GAPDH (Cell Signaling Technology 5174), and β-actin (Cell Signaling Technology 3700S).

### ISH.

ISH was performed as previously described (3). Mouse Foxm1 riboprobes: 5′ → 3′ Sense: CATTTAGGTGACACTATAGGCTATCCAACTCCTGGGAAGATTC. 5′ → 3′ Antisense: TAATACGACTCACTATAGGGCAATGTCTCCTTGATGGGGGTC. The riboprobe sequence was adapted (71).

### Cell culture experiments.

Primary human proximal tubular cells were purchased from Lonza (CC-2553) and cultured with REGM Renal Epithelial Cell Growth Medium BulletKit (CC-3190 Lonza). Cells were maintained in a humidified 5% CO_2_ atmosphere at 37°C. Experiments were carried out on early-passage cells. RNA was extracted using the MiniPrep Kit (QIAGEN). Inhibitors U0126, SCH772984, rapamycin, PF-0421790, and LY2109761 were bought from MedChemExpress; diluted in DMSO; and stored at –80°C until use. TGF-β, HGF, and EGF were from Proteintech. For the inhibitor treatments, the cells were serum-starved overnight and then treated with the inhibitor for 24 hours. For the ligand-receptor experiments, the cells were initially treated for 1 hour with the inhibitor only, then cultured with both the ligand and the inhibitor for 24 hours.

### mPTEC isolation.

Primary mouse tubular epithelial cells were isolated from 8- to 10-week-old *Foxm1^fl/fl^* mice using a previously published protocol (72) with slight modifications. Briefly, kidney cortex was isolated by removing the medulla. It was then minced and digested for 30 minutes at 37°C in a solution with Collagen IV (Worthington). The cell suspension was neutralized and filtered through a 70 μM filter (Falcon, Corning). The cells were washed once with Dulbecco’s PBS and then resuspended in fresh DMEM/F12 medium containing 1× ITS (insulin, transferrin, selenium), penicillin/streptomycin, hydrocortisone (40 μg/mL), and murine EGF (10 ng/mL).

### Adenovirus transduction.

For *Foxm1* knockout, mPTECs from *Foxm1^fl/fl^* mice were isolated and infected with Ad5CMV-EGFP (control) or Ad5CMVCre-EGFP (University of Iowa Viral Vector Core) at 50 MOI for 24 hours. Infection efficiency was monitored under fluorescence microscope (Leica DM IL LED) by GFP expression.

### FOXM1 and CCNF siRNA transfection.

hRPTECs were grown to 50%–60% confluence, at which point they were transfected with 10 nmol/L of target siRNA (Foxm1 5248 or CCNF 2528) or negative control siRNA (Silencer Select, Thermo Fisher Scientific) using Lipofectamine RNAiMAX (Life Technologies, Thermo Fisher Scientific) following the manufacturer’s protocol. Cells were harvested at day 2 posttransfection for protein and RNA isolation to validate knockdown.

### MTS assay.

hRPTECs were transfected with CCNF siRNA or negative control as above. Cells were seeded at a density of 1,250 cells per well in a 96-well plate in renal epithelium cell growth medium (Lonza) with 4 replicates per group. CellTiter 96 AQueous One Solution Cell Proliferation Assay (Promega) was used as per manufacturer’s protocol. Optical density readings were taken 2 hours after first seeding for day 0 and subsequently on day 1, 2, 3, and 4.

### CUT&RUN.

CUT&RUN assays for determination of *FOXM1* binding sites in hRPTECs were performed with CUTANA kit (EpiCypher, 14-1048) based on the manufacturer’s instructions. The primary hRPTECs with early passages (P4) were seeded at 1 × 10^6^ cells per 10 cm culture dish 24 hours prior to the assay. The cells were fixed with 0.5% formaldehyde (MilliporeSigma, 25259) for 1 minute at room temperature, and fixation reaction was quenched by adding glycine to a final concentration of 125 mM. Subsequently, the cells were scraped from culture dishes and centrifuged at 500*g* for 5 minutes. Pellets were resuspended in PBS with 1% BSA and counted. The cells were then centrifuged at 500*g* for 5 minutes and resuspended with wash buffer provided in the CUTANA kit. A total of 500,000 cells in 100 μL wash buffer were mixed and incubated with Concanavalin A–conjugated paramagnetic beads, followed by addition of antibodies to each sample (0.5 μg of FOXM1 antibody [Cell Signaling Technology, 5436, 1:50] or rabbit IgG negative control antibody [EpiCypher, 13-0041, 1:50]). The remaining steps were performed with the manufacturer’s instructions for cross-linked samples. Library preparation was performed using the NEBNext Ultra II DNA Library Prep Kit for Illumina (New England Biolabs, E7645S) according to the manufacturer’s instructions, including minor modifications indicated by the CUTANA kit described above. The CUT&RUN libraries were sequenced with NovaSeq (Illumina, 150 bp paired-end reads). The FASTQ files were trimmed with Trim Galore (Cutadapt v2.8) and aligned with Bowtie2 (v2.3.5.1) (parameters: --local --very-sensitive-local --no-unal --no-mixed --no-discordant --phred33 -I 10 -X 700) using hg38 reference genome. The peaks were detected by MACS2 (v2.2.7.1) with default parameters (macs2 peakcall). The BAM files were converted to BigWig format with bamCoverage (deepTools 3.5.0) and subsequently visualized by Integrative Genomics Viewer (v2.9.4). The consensus list of FOXM1 peaks among the triplicate data sets was generated using the intersect function in bedtools (v2.27.1).

### CRISPRi.

sgRNAs targeting *CCNF* putative enhancer regions or promoters were designed using CHOPCHOP (https://chopchop.cbu.uib.no/) (73). Sequences of the sgRNAs are as follows: CCNF_enhancer_1-1, 5′-GCGCCACGTTTCGCGGGAAGA-3′; CCNF_enhancer_1-2, 5′-GAGCAGATACGACACTTCCCG-3′; CCNF_enhancer_2-1, 5′-GTTCCTGTGCCTCAACGCGCG-3′; CCNF_enhancer_2-2, 5′-GTCGTCGCCCTGGAATACGTT-3′; CCNF_promoter-3, 5′-GCGGCGAAGCCCGAACCCATG-3′.

The sgRNAs were inserted downstream of the U6 promoter of the dCas9-KRAB repression plasmid (pLV hU6-sgRNA hUbC-dCas9-KRAB-T2a-Puro, 71236). Briefly, sense and antisense oligonucleotides were annealed by cooling from 95°C to 25°C for 1.5 hours. The annealed oligonucleotides were then subcloned into the dCas9-KRAB repression plasmid by Golden Gate Assembly with Esp3I restriction enzyme (New England Biolabs, R0734L) and T4 DNA ligase (New England Biolabs, M0202L) on a thermal cycler repeating 37°C for 5 minutes and 16°C for 5 minutes for 60 cycles, followed by transformation to NEB 5-alpha Competent *E*. *coli* (New England Biolabs, C2987H) as per manufacturer’s instructions. The cloned lentiviral vectors were purified with QIAprep Spin Miniprep Kit (QIAGEN, 27106) or PureLink HiPure Plasmid Midiprep Kit (Thermo Fisher Scientific, K210004), and sgRNA insertion was confirmed with Sanger sequencing by GENEWIZ.

To generate lentivirus, HEK293T cells (ATCC, CRL-3216) were seeded at 1.2 × 10^6^ cells on a 6 cm tissue culture dish 1 day before transfection. The cells were transfected with 3.5 μg of psPAX2 (Addgene, 12260), 0.35 μg of pMD2.G (Addgene, 12259), and 3.5 μg of dCas9-KRAB repression plasmid with sgRNAs by Lipofectamine 3000 transfection reagent (Thermo Fisher Scientific, L3000015) as per the manufacturer’s instructions. Culture media were changed to DMEM supplemented with 30% FBS at 24 hours after transfection. Lentivirus-containing supernatants were collected 24 and 48 hours later and filtered through 0.45 μm PVDF filters (CELLTREAT, 229745).

For transduction to hRPTECs, cells were seeded at 5.0 × 10^4^ cells per well on 6-well tissue culture plates 1 day before transfection. Culture media were then changed to the lentiviral supernatants supplemented with 3 μg/mL polybrene (Santa Cruz Biotechnology, sc-134220) and transduced for 24 hours. At 48 hours from transduction, transduced cells were selected by 3 μg/mL of puromycin (InvivoGen, ant-pr-1) for 72 hours and collected for analysis.

### Bulk RNA-Seq analysis.

Samples were prepared according to library kit manufacturer’s protocol, indexed, pooled, and sequenced on an Illumina NovaSeq 6000. Base calls and demultiplexing were performed with Illumina’s bcl2fastq2 software. RNA-Seq reads were then aligned and quantitated to the Ensembl release 101 primary assembly with an Illumina DRAGEN Bio-IT on-premise server running version 3.9.3-8 software. DEG analysis was performed using the edgeR package (74) and setting a cutoff counts per million of more than 0.4 and FDR of less than 5%. Gene Ontology analysis was performed using DAVID (75) and analyzed using the functional annotation tool.

### Statistics.

Data are presented as mean ± SEM. Unpaired 2-tailed Student’s *t* test was used to compare 2 groups, and *P* value of less than 0.05 was considered significant. For multiple-group comparisons, 1-way or 2-way ANOVA followed by post hoc correction with Dunnett’s test or Bonferroni’s test where appropriate was applied. Statistics were performed using Prism 10.0 (GraphPad Software).

### Study approval.

All mouse experiments were approved by the Animal Care and Use Committee at Washington University in St. Louis.

### Data availability.

RNA-Seq data and CUT&RUN data were deposited in the NCBI’s Gene Expression Omnibus database under accession number GSE234444. ATAC-Seq data in primary hRPTECs were previously published (57) and are available under accession number GSE195443. CUT&RUN sequencing data for H3K4me3 and H3K27ac in primary hRPTECs are published (58) and are publicly available under GSE220289. Values for all data points in graphs are reported in the Supporting Data Values file.

## Author contributions

MLN and MCP performed experiments with contributions from YM, YY, and ALH. YM and HW performed bioinformatic analysis and analyzed data with MCP. VVK provided reagents. MCP and BDH conceived of the work, designed experiments, analyzed results, and wrote the manuscript with MLN.

## Supplementary Material

Supplemental data

Unedited blot and gel images

Supporting data values

## Version 1 06/25/2024

In-Press Preview

## Version 2 08/08/2024

Electronic publication
