# Supplementary material for: Injury-induced Foxm1 expression in the mouse kidney drives epithelial proliferation by a cyclin F–dependent mechanism
Source: JCI Insight. 2024 Jun 25;9(15):e175416. doi: 10.1172/jci.insight.175416 (PMC11383596; doi:10.1172/jci.insight.175416)
Supplement: Supplemental data [file jciinsight-9-175416-s089.pdf]

# Injury-induced FoxM1 expression in mouse kidney drives epithelial proliferation by a Cyclin F dependent mechanism

Megan L. Noonan, Yoshiharu Muto, Yasuhiro Yoshimura, Aidan Leckie-Harre, Haojia Wu, Vladimir V. Kalinichenko, Benjamin D. Humphreys, and Monica Chang-Panesso

**Supplemental Information**

Supplemental Figure 1.

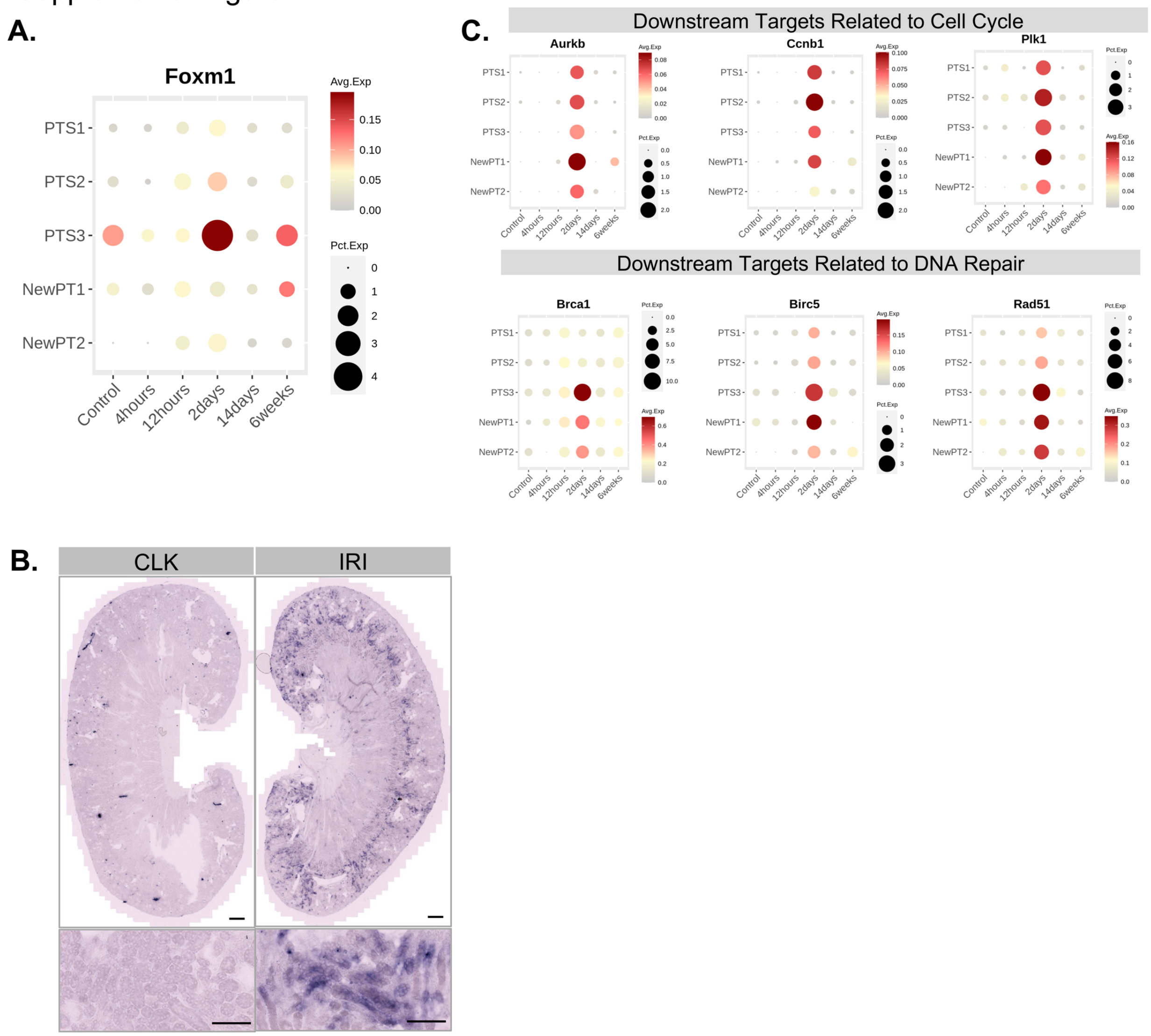


**SUPPLEMENTAL FIGURE1.**

***Foxm1* upregulation is detected in single nucleus RNA sequencing of mouse injured kidneys. (A)** *Foxm1* expression in a previously published dataset (1) showing upregulation at day 2 with predominance in the S3 segment of the proximal tubule. **(B)** Foxm1 expression by in situ hybridization in kidney sections after 2 days of unilateral IRI. CLK: contralateral, IRI: ischemia reperfusion injury. Scale bars: 500 µM (upper panels); 50 µM (lower panels)**. (C)** *Foxm1* downstream targets related to cell cycle and DNA repair are also upregulated at day 2 after ischemic injury in a previously published single nucleus RNA sequencing study (1).

Supplemental Figure 2.

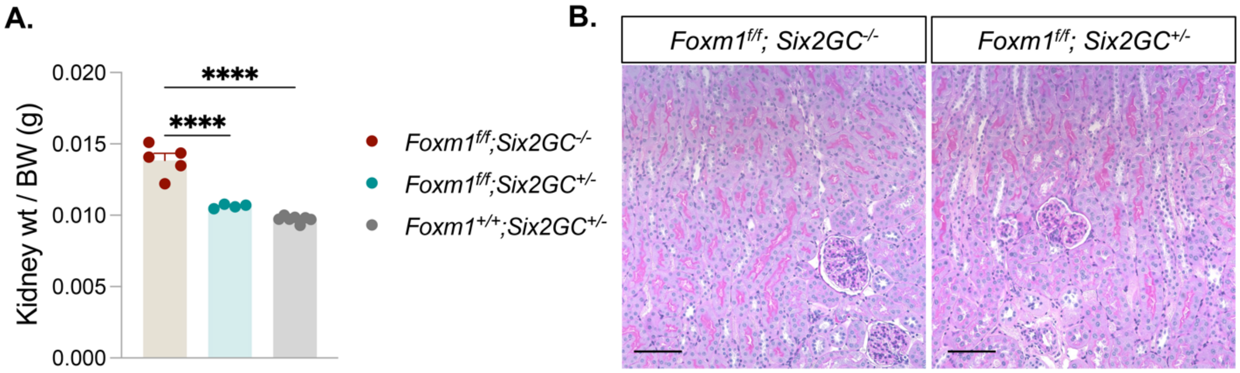


**SUPPLEMENTAL FIGURE 2.**
**(A)** Kidney weight in relation to body weight from three different mouse lines. **(B)** PAS staining of kidney sections from sham mice. For A*, n*=4-7, ****$P$ <0.0001 by one-way ANOVA with post-hoc Dunnet's multiple comparison test. Scale bar 100 μM.

Supplemental Figure 3.

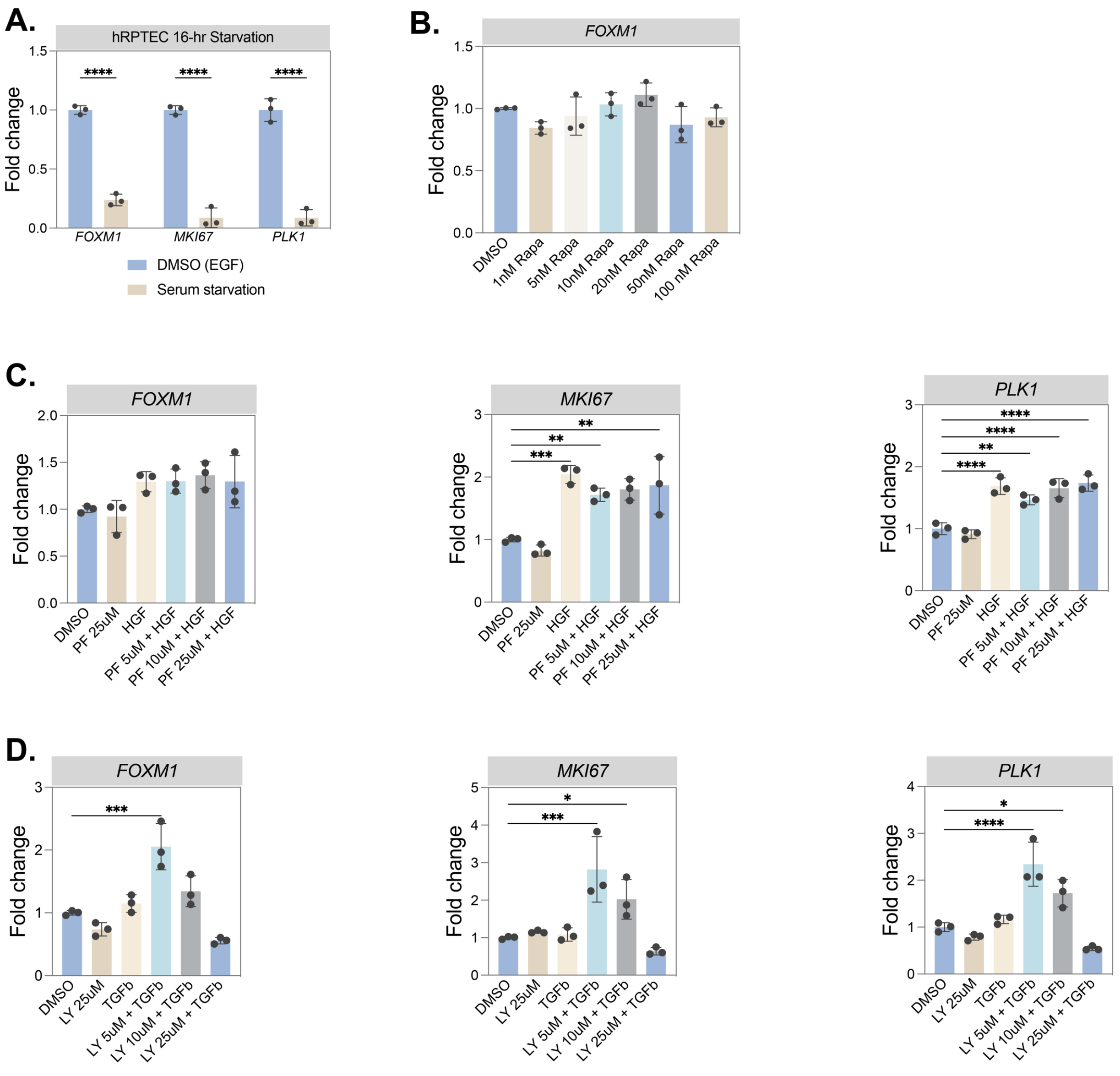


**SUPPLEMENTAL FIGURE 3.**

***FOXM1* mRNA expression is unaffected by mTOR or c-Met receptor inhibition**. **(A)** *FOXM1* is downregulated in primary hRPTECs after overnight serum starvation. **(B)** *FOXM1* mRNA expression after treatment of primary hRPTECs for 24 hours with Rapamycin at various concentrations. *FOXM1*, *MKI67*, and *PLK1* mRNA expression in primary hRPTECs after 24-hour treatment with either c-met inhibitor, PF-04217903 **(C)** or TGFβ I/II receptor inhibitor, LY2109761 **(D)** HGF 10 ng/mL and TGF-β 2 ng/mL. For A-D*, n*=3. *P <0.05, **P < 0.01, ***P<0.001, *****P* <0.0001 by two-way ANOVA with post-hoc Sidak's multiple comparison test in (A) and one-way ANOVA with post-hoc Dunnet's multiple comparison test in (B-C).

Supplemental Figure 4.

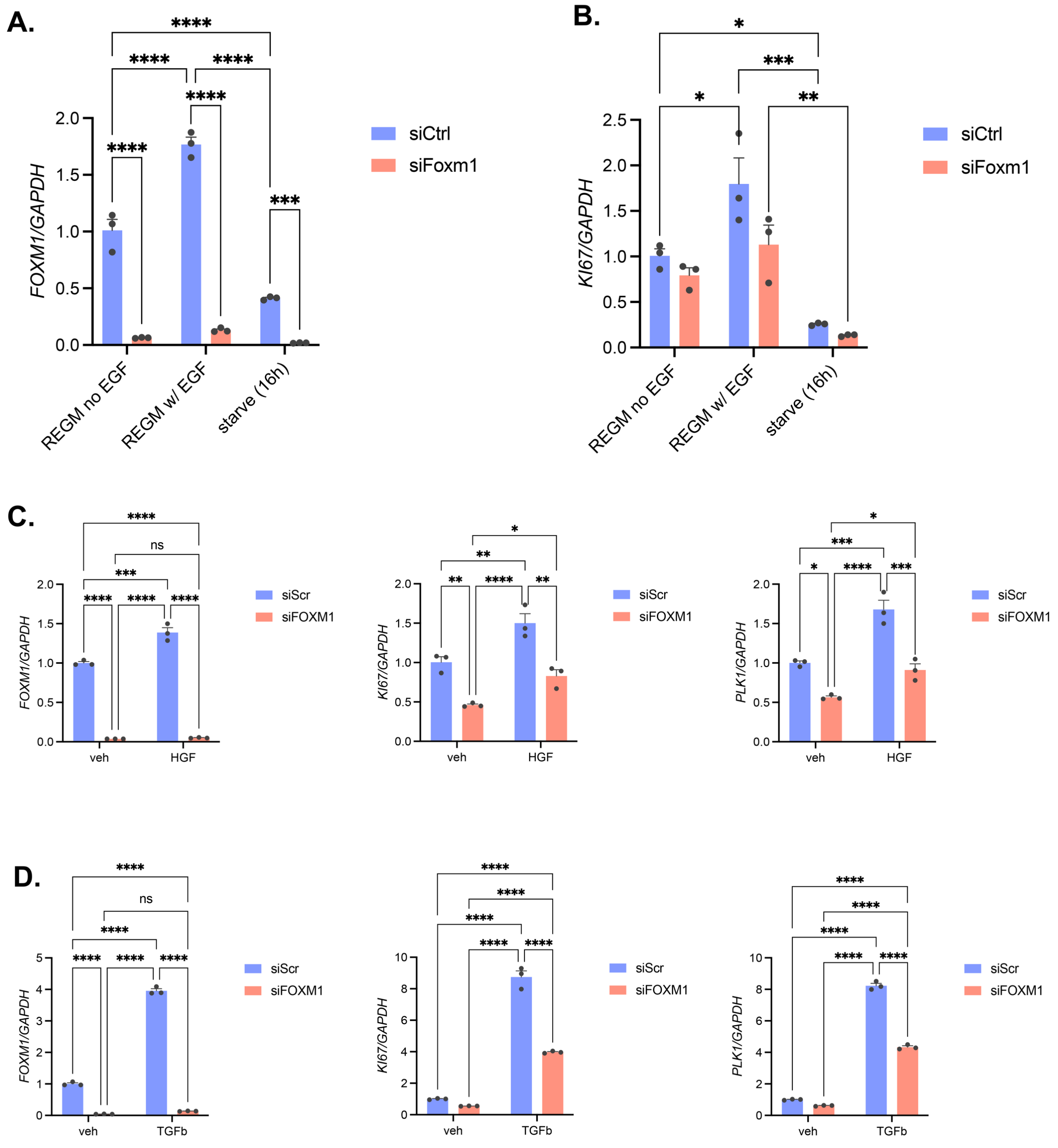


**SUPPLEMENTAL FIGURE 4.**

***FOXM1* knockdown ameliorates EGF proliferative response, but not with HGF or TGF-β treatments.** *FOXM1* **(A)** and *MKI67* **(B)** expression in primary human RPTECs transfected with siRNA against *FOXM1* (siFOXM1) or a scrambled control siRNA (siScr) for 24 hours, then cultured with and without the standard EGF component of the REGM media for 24 hours or after 16 hours of overnight serum starvation. *FOXM1*, *MKI67*, and

*PLK1* mRNA expression in hRPTECs with *FOXM1*-knockdown (siFOXM1), then treated with either HGF **(C)** or TGF-β **(D)**. HGF 10 ng/mL and TGF-β 2 ng/mL. For A-D, n=3. *P <0.05, **P < 0.01, ***P<0.001, *****P* <0.0001 by two-way ANOVA with post-hoc Tukey's multiple comparison test.

Supplemental Figure 5.

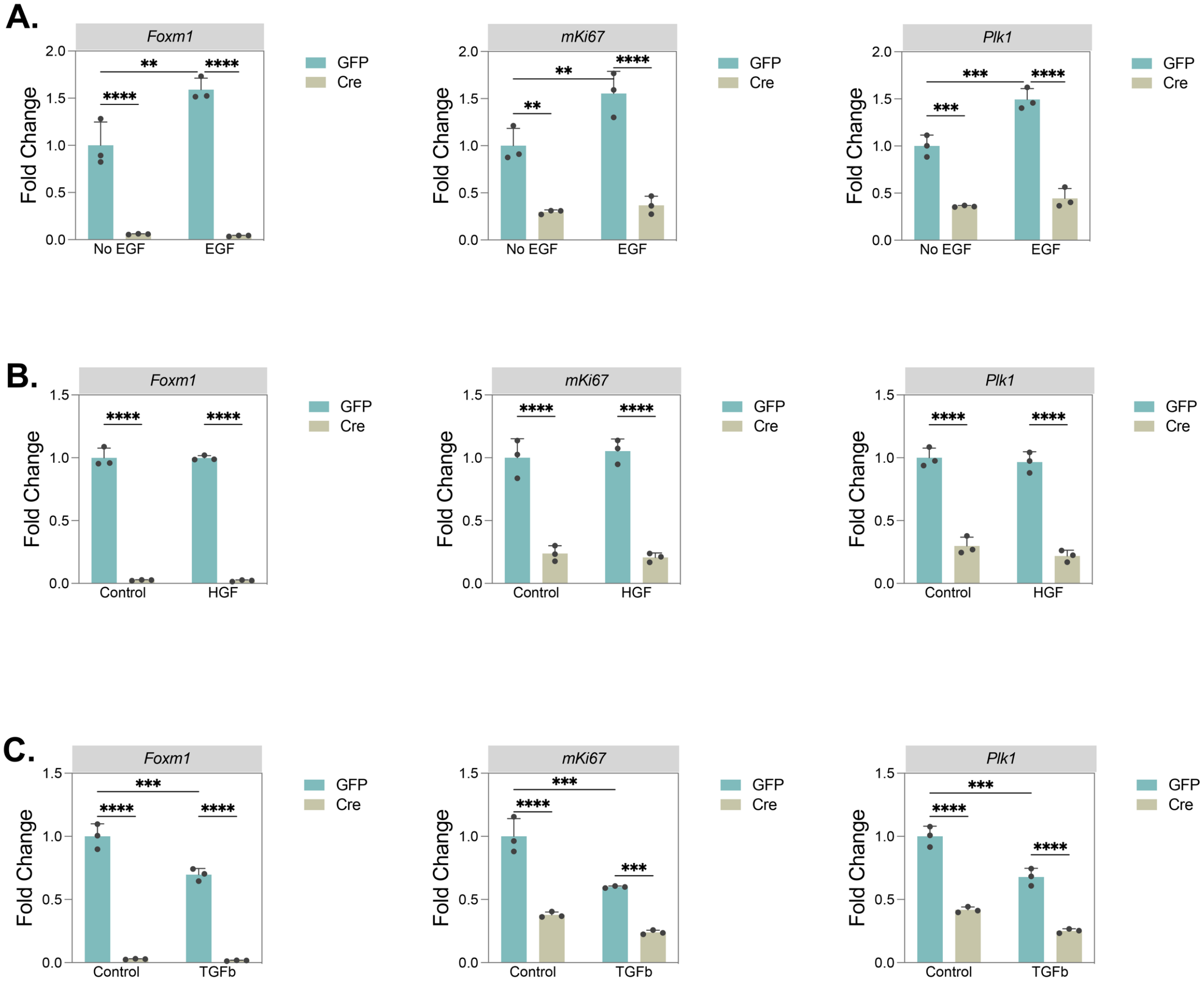


**SUPPLEMENTAL FIGURE 5.**

**EGF induces *Foxm1* upregulation in primary mouse RPTECs, but *Foxm1* remains unchanged with HGF stimulation and is downregulated with TGFβ treatment.** Primary mouse RPTECs from Foxm1 f/f mice were transduced with either Adeno-Cre virus or Adeno-GFP virus control and treated for 24 hours with the specified ligands. *Foxm1*, *mKi67*, and *Plk1* mRNA expression in primary mouse RPTECs after 24-hour treatment with either EGF **(A)**, HGF **(B)**, or TGFβ **(C)**. HGF 10 ng/mL and TGFβ 2 ng/mL. For A-C, n=3. *P <0.05, **P < 0.01, ***P<0.001, ****$P$ <0.0001 by two-way ANOVA with post-hoc Sidak's multiple comparison test.

Supplemental Figure 6.

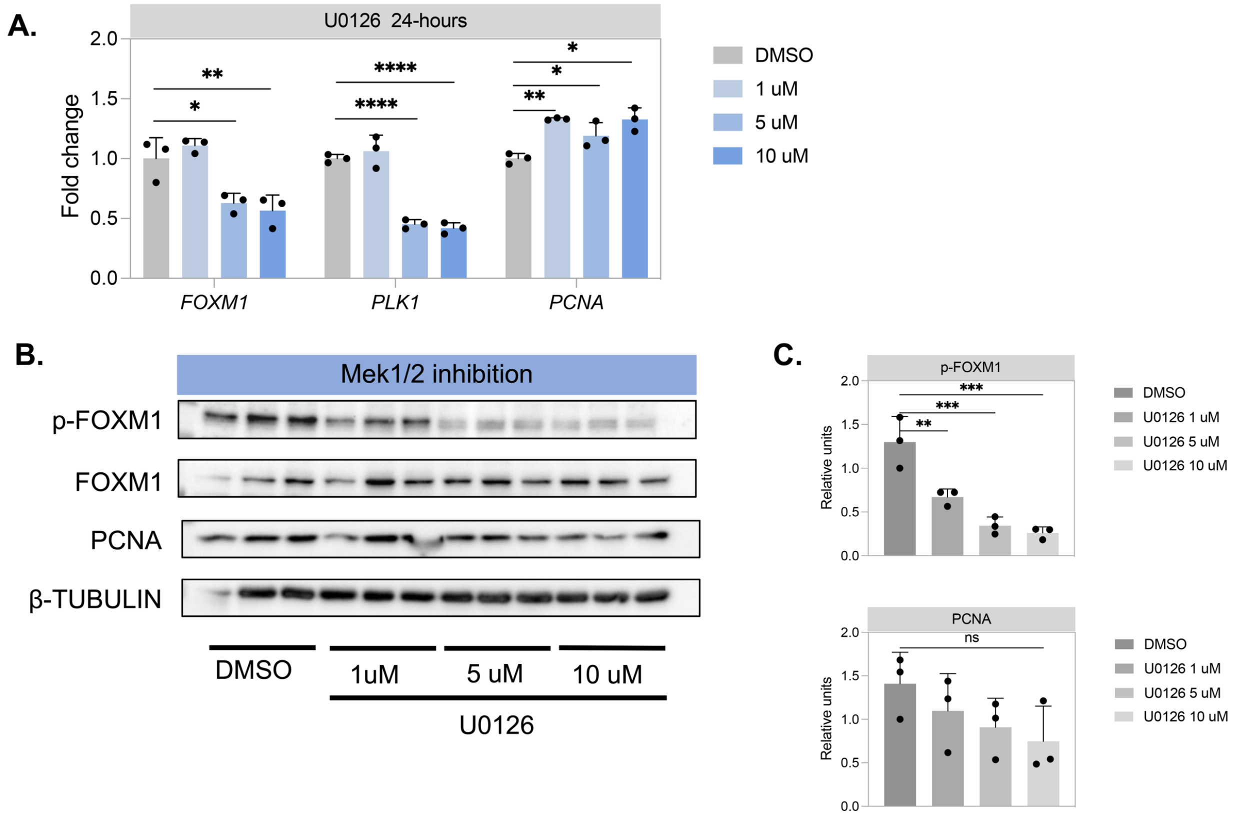


**SUPPLEMENTAL FIGURE 6.**
***MEK* signaling upstream of *FOXM1*. (A)** *FOXM1*, *PLK1* and *PCNA* mRNA expression by qPCR in cell lysates from hRPTECs treated for 24 hrs with various doses of the MEK inhibitor U0126. **(B)** Western blot from cell lysates treated with U0126 and **(C)** densitometry of the phospho-FOXM1 and PCNA bands in (B). For all experiments, n=3 replicates per group. **P* < 0.05, ***P* < 0.01; ****P* < 0.001, *****P*<0.0001 by one-way ANOVA with post-hoc Dunnet's multiple comparison test.

**Supplemental Table 1**

**Mouse Primers**

| Gene | Forward primer 5’ -> 3’ | Reverse primer 5’ -> 3’ |
|---|---|---|
| *Foxm1* | GGA CAT CTA CAC TTG GAT TGA GG | TGT CAT GGA GAG AAA GGT TGT G |
| *Plk1* | ACC TAC CTC CGG ATC AAG AAA | AGA ACT CGT CAT TGA GCA ACT C |
| *PCNA* | GCT TGG CAA TGG GAA CAT TAA G | CGT TAG GTG AAC AGG CTC ATT |
| *Mki67* | GAA GTC TCT TGG CAC TCA CA | GCG TCT TTG ATC ATT TGT CCT C |
| *Acta2* | CTG ACA GAG GCA CCA CTG AA | CAT CTC CAG AGT CCA GCA CA |
| *Fn* | ATG TGG ACC CCT CCT GAT AGT | GCC CAG TGA TTT CAG CAA AGG |
| *Vcam1* | CTC TTA CCT GTG CGC TGT GA | GGA TCT TCA GGG AAT GAG TAG ACC |
| *Ccnf* | GGG TGC TAA ATC TTT TTG AGG ACG | ACC TTT CTG TCG CTT TCC CA |
| *Rrm2* | AGG CTA CGT ATG GAG AAC GC | ATC AGC CCC CGT TTC TTG AG |
| *E2f1* | CCA CGA GGC CCT TGA CTA TC | ACA CCC TGA ATC CCT AGG CT |
| *Gapdh* | AGG TCG GTG TGA ACG GAT TTG | TGT AGA CCA TGT AGT TGA GGT CA |

**Human Primers**

| Gene | Forward primer 5’ -> 3’ | Forward primer 5’ -> 3’ |
|---|---|---|
| *FOXM1* | AAA GGA GAA TTG TCA CCT GGA G | TGG CCA TGT AAG AGT AGG GT |
| *PLK1* | CAC AGT TTC GAG GTG GAT GT | ATC CGG AGG TAG GTC TCT TT |
| *PCNA* | CAG ACT ATG AAA TGA AGT TGA TGG A | CGT GCA AAT TCA CCA GAA GG |
| *CCNF* | CCC GAA ACC TGA CCA TCT TGA | ACA CAC TGG CGT GGT TGT C |
| *PCNA* | CAG ACT ATG AAA TGA AGT TGA TGG A | CGT GCA AAT TCA CCA GAA GG |
| *MKi67* | CTT TGG GTG CGA CTT GAC GA | ACA ACT CTT CCA CTG GGA CG |
| *GAPDH* | GAC AGT CAG CCG CAT CTT CT | GCG CCC AAT ACG ACC AAA TC |

**References:**

1. Kirita Y, et al. Cell profiling of mouse acute kidney injury reveals conserved cellular responses to injury. *Proc Natl Acad Sci U S A*. 2020;117(27):15874–15883.
