# Supplementary material for: Injury-induced Foxm1 expression in the mouse kidney drives epithelial proliferation by a cyclin F–dependent mechanism
Source: JCI Insight. 2024 Jun 25;9(15):e175416. doi: 10.1172/jci.insight.175416 (PMC11383596; doi:10.1172/jci.insight.175416)

Full unedited gel for Figure 2F.

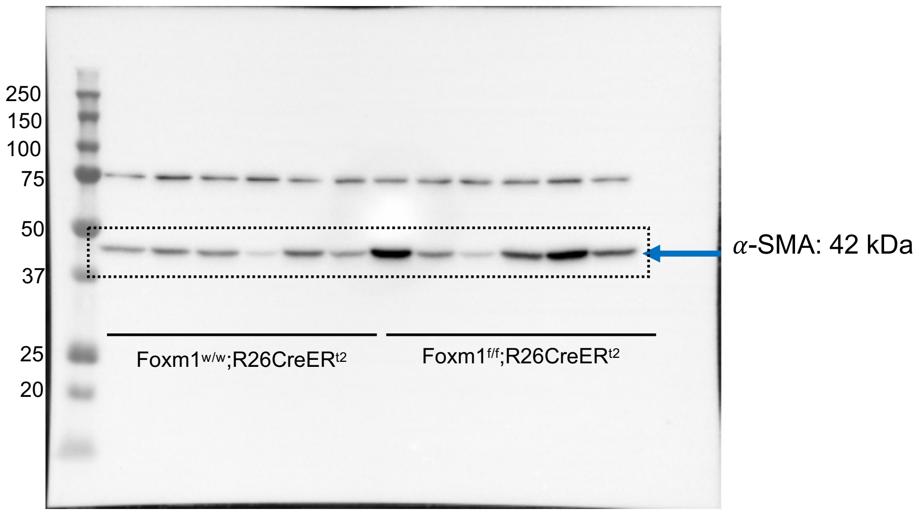

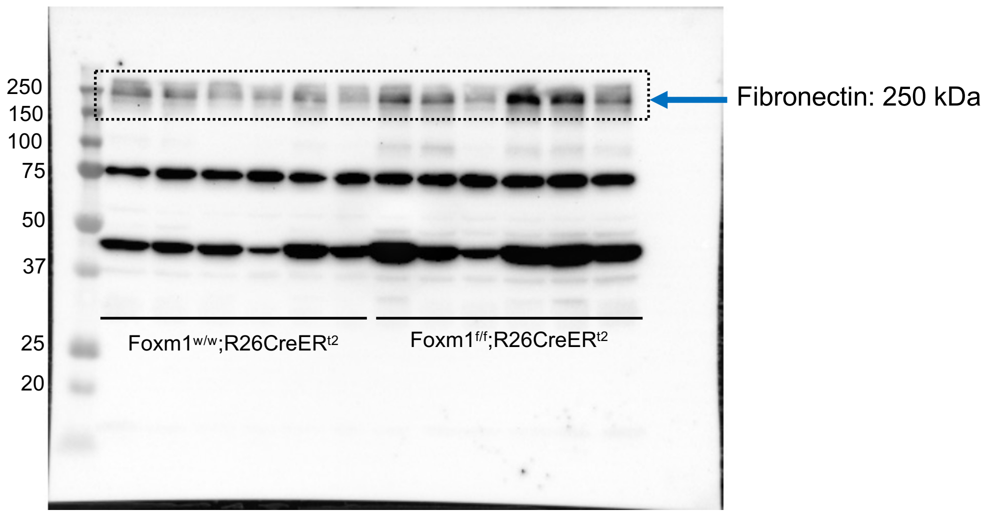

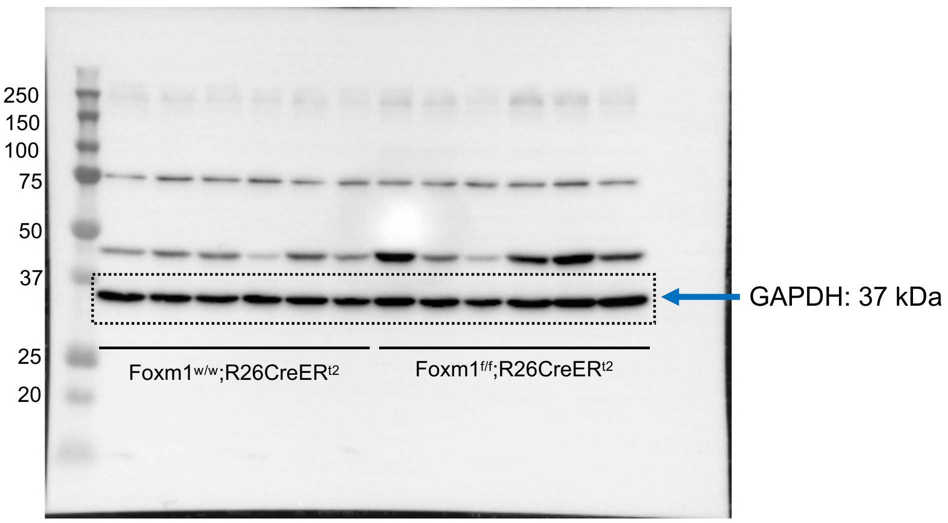

Full unedited gel for Figure 3D.

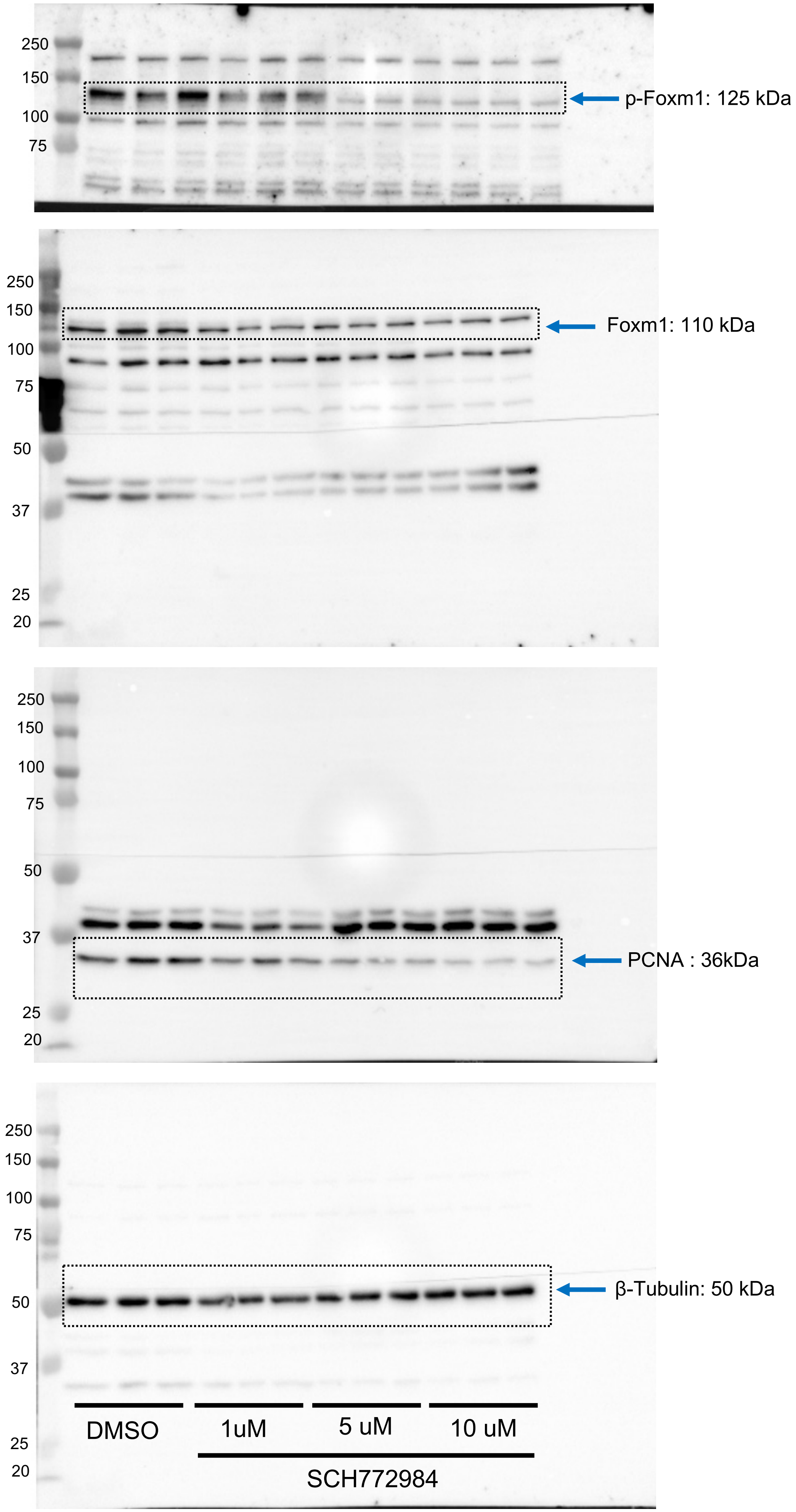

Full unedited gel for Supplemental Figure 6.

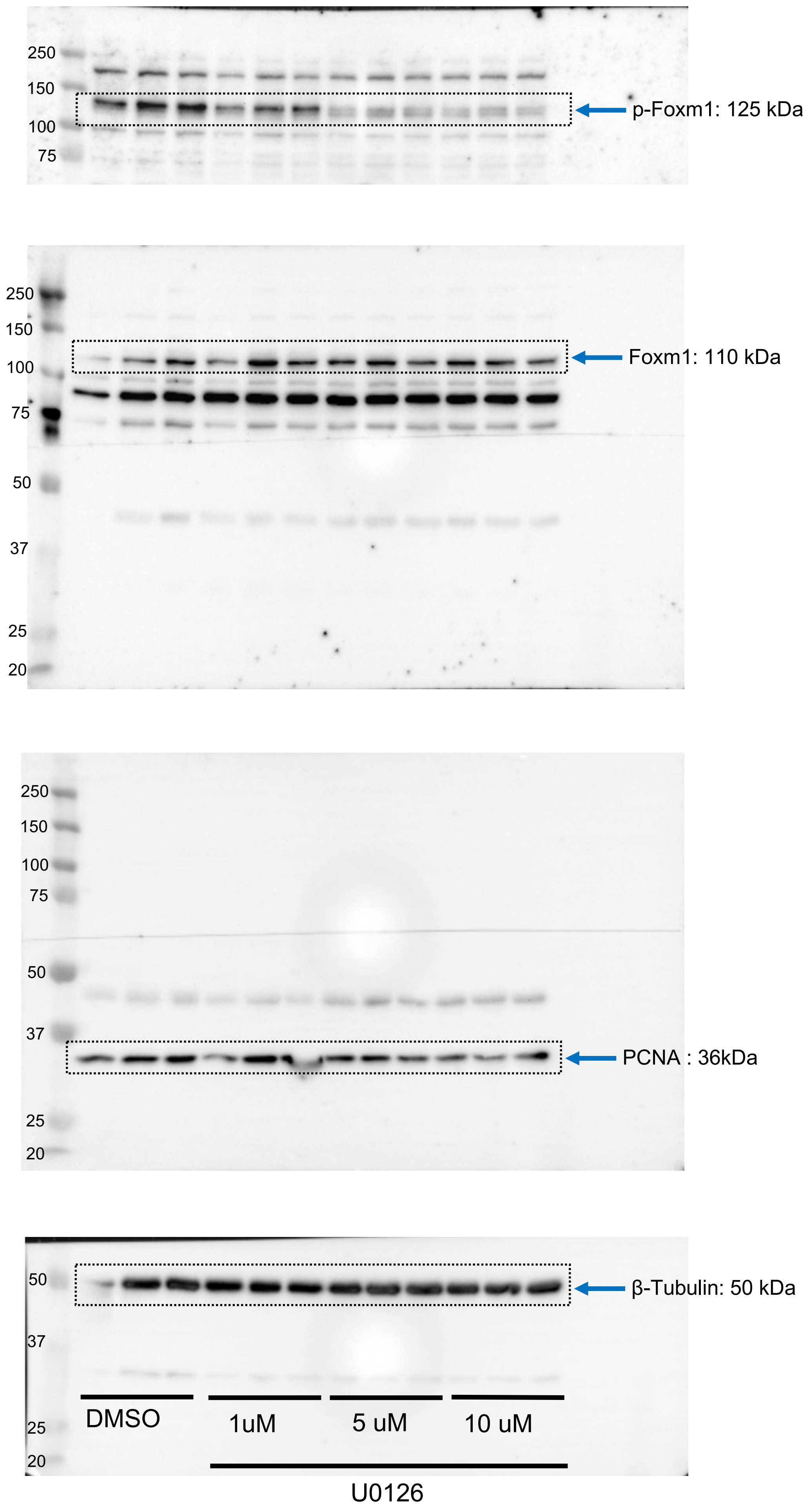

Supplement: Unedited blot and gel images [file jciinsight-9-175416-s090.pdf]
